# Why Antibias Interventions (Need Not) Fail

**DOI:** 10.1177/17456916211057565

**Published:** 2022-05-17

**Authors:** Toni Schmader, Tara C. Dennehy, Andrew S. Baron

**Affiliations:** Department of Psychology, University of British Columbia

**Keywords:** implicit bias, stereotyping and prejudice, diversity training, microaggressions

## Abstract

There is a critical disconnect between scientific knowledge about the nature of bias and how this knowledge gets translated into organizational debiasing efforts. Conceptual confusion around what implicit bias is contributes to misunderstanding. Bridging these gaps is the key to understanding when and why antibias interventions will succeed or fail. Notably, there are multiple distinct pathways to biased behavior, each of which requires different types of interventions. To bridge the gap between public understanding and psychological research, we introduce a visual typology of bias that summarizes the process by which group-relevant cognitions are expressed as biased behavior. Our typology spotlights cognitive, motivational, and situational variables that affect the expression and inhibition of biases while aiming to reduce the ambiguity of what constitutes implicit bias. We also address how norms modulate how biases unfold and are perceived by targets. Using this typology as a framework, we identify theoretically distinct entry points for antibias interventions. A key insight is that changing associations, increasing motivation, raising awareness, and changing norms are distinct goals that require different types of interventions targeting individual, interpersonal, and institutional structures. We close with recommendations for antibias training grounded in the science of prejudice and stereotyping.

Events in recent years have initiated a collective conversation about how our social, corporate, and governmental institutions approach issues of diversity, equity, and systemic bias. The #MeToo movement increased awareness of sexual harassment, mass grave sites at Canada’s residential schools drew greater attention to the genocide of indigenous peoples, and worldwide protests in 2020 called for action to address persistent racial injustice. What form said action should take has been subject to some debate; efforts to change policies and structures that contribute to systemic biases have at times been pitted against educational efforts to eradicate (implicit) biases in the minds and actions of individuals. As antibias trainings are easier to implement than structural change, it is perhaps unsurprising that they have risen in popularity. Increasingly, those in positions of power, including police officers, educators, C-level executives, and hiring managers, are asked to undergo training aimed to make them less biased. The stated purpose of many trainings is to raise employees’ awareness of *unconscious* or *implicit* bias, under the assumption that such bias need only be brought into awareness to be vanquished.

Alongside the proliferation of antibias trainings, people have expressed skepticism that such trainings may be ineffective or even counterproductive (e.g., Green & Hagiwara, 2020). Political opposition to antibias trainings has also grown; for example, in September 2020, former U.S. president Donald Trump issued an executive order banning many forms of diversity training as being anti-American (Exec. Order No. 13,950, 2020). Antibias trainings—and whether they are necessary or effective—have become a “hot-button” issue, one that psychological science is ideally positioned to address.

Despite advances in our scientific understanding of how and when stereotypes and prejudiced attitudes can shape judgment and behavior, there is a profound gap between this research and its practical application. This gap is exacerbated by a common misunderstanding of what implicit bias is and a failure to distinguish between *reducing* the implicit associations people have formed and *regulating* the degree to which those associations influence behavior. A primary objective of this article is to introduce a heuristic typology that articulates how biased outcomes result from implicit or explicit processes that are distributed among individuals in a social context. This typology then allows us to distinguish between implicit and explicit (or intentional) forms of biased behavior. Why should this matter? As scientists, a common typology and set of terms afford greater precision in comparing findings and approaches across lines of research. For practitioners, we need greater conceptual clarity in operationalizing interventions aimed at counteracting bias.

Toward these aims, we first begin with a critical review of some pitfalls of existing antibias training before providing a primer on how dual-process approaches bring greater clarity to the nature of bias. Our typology will reveal key distinctions between different forms of biased expression, enabling practitioners and theoreticians to identify distinct bias pathways as well as critical entry points to improve the efficacy of antibias trainings. Our focus on targeting interventions to counteract different pathways to bias is intended to complement other recent discussions of how best to mitigate bias in organizations (Carter et al., 2020).

## Flawed by Design: Six Pitfalls of Antibias Training

The public appetite for evidence-based solutions to mitigating bias has never been larger. McKinsey estimated in 2017 that $8 billion dollars are spent each year by companies on some form of antibias training (Kirkland & Bohnet, 2017). That number is likely to be larger in the wake of 2020’s mass protests for racial justice and the #MeToo movement. In light of this increasing public and corporate commitment to antibias goals, it is imperative that we correct any misapplications of psychological science that can undermine the effectiveness of antibias training. In short, we believe antibias trainings are often flawed by design because of several pitfalls of existing approaches both in the field and in the lab (see Table 1).

**Table 1.** Six Pitfalls of Antibias Training

1. Antibias training is rarely subjected to rigorous peer-reviewed research aimed at evaluating and improving the effectiveness of such interventions.
2. Antibias training is not always conducted with broad organizational buy-in or the assurance that management and employees have a genuine motivation to foster inclusion.
3. Antibias training too often assumes that the primary objective is to change people’s implicit associations (stereotypes or attitudes).
4. Antibias training is not always constructed with a clear definition of what implicit bias is or grounded in the science of how bias unfolds.
5. Antibias training too often assumes that making people aware of their own stereotypes or prejudices will eliminate biased behavior.
6. Antibias training often focuses on educating an individual without considering the broader cultural context in which the individual lives, works, or learns.

First, less than 1% of all research on the topic of prejudice reduction uses experimental methodology carried out with adults in field-based settings (Paluck et al., 2020). Most of the training in organizations is conducted by private consulting firms or resident diversity specialists who might have no expertise on the science of bias (Zelevansky, 2019). The dearth of published research suggests the evaluation of antibias training is rarely shared or submitted for independent review. Thus, the first critical misstep of antibias training is the failure to conduct rigorous peer-reviewed research aimed at evaluating and improving the effectiveness of these trainings in the field.

Second, academic research paints a less than optimistic picture about the potential for antibias training to create organizational change. This may in part be due to organizations’ desire to merely reduce their legal liability in discrimination lawsuits or to advertise claimed values to consumers without necessarily fostering broader inclusion and diversity among staff. After all, people assume that companies with diversity policies in place are less likely to discriminate against employees (Kaiser et al., 2013). Dobbin and Kalev (2016) reported that although voluntary training programs can be somewhat effective in boosting the hiring rates of ethnic minorities into management positions, these efforts can spark backlash when training is made mandatory. Their research revealed a 5% decrease in Asian women and a 9% decrease in Black women in management positions over 5 years when diversity trainings were made mandatory. In another meta-analysis offering a more optimistic perspective, Bezrukova et al. (2016) suggested that the backlash to mandatory training may be limited to people’s attitudes toward the training itself given evidence that training can actually debias behavior. Nonetheless, an ideal approach would foster equity, diversity, and inclusion without inciting large-scale backlash (Emerson, 2017). Therefore, the second pitfall of antibias training is failing to cultivate genuine motivation to foster inclusion by either management or employees.

Third, antibias trainings often have a narrow goal to change people’s implicit associations (specifically their stereotypes and attitudes). This peculiar focus is a by-product of the popular attention received by the Implicit Association Test (IAT; Greenwald et al., 1998) and Project Implicit (https://implicit.harvard.edu/implicit/). By using sets of speeded categorizations, the IAT assesses the strength of one’s cognitive association between two categories in a way that is distinct from self-reported beliefs and attitudes. Although the IAT has been validated as a measure of individual difference (Greenwald et al., 2009), too often the terms “implicit associations” (the strength of the associations between concepts in the mind, measured indirectly by the IAT) and “implicit bias” (disparate treatment that can result from one’s implicit associations with social groups) are used isomorphically (cf. De Houwer, 2019). This conceptual confusion creates the impression that changing implicit associations is the best way to reduce implicit bias. However, research offers little hope that this is feasible. Although some manipulations reduce implicit negative associations with African Americans (Lai et al., 2014), these effects are short-lived (Lai et al., 2016), and Baron (2015) suggested that by adulthood it may be too late to efficiently change these associations. Thus, antibias trainings should not maintain a dominant focus on changing underlying implicit associations when biased behavior, as we discuss below, is driven by a multitude of factors. The third pitfall of antibias training is to assume that the primary objective should be to change people’s implicit associations.

The fourth pitfall, related to the last, is a fundamental lack of clarity on what implicit bias is. Terms such as implicit bias, implicit associations, unconscious bias, systemic bias, and microaggressions are often used interchangeably in public discourse with little understanding of the scientifically grounded process by which bias unfolds. This confusion is not limited to the public; theoreticians also debate the meaning of these terms. Recent critiques highlight the vagueness and utility of the term “microaggression” (Lilienfeld, 2017); there is also a push to move beyond understanding implicit bias as an individual-difference variable and toward viewing it as a reflection of the broader social context (De Houwer, 2019; Gawronski, 2019; Payne et al., 2017). One of our goals is to expand these debates by outlining how a clear conceptualization of bias as an outcome resulting from a distributed process can inform antibias interventions. To that end, we define implicit bias as the disparate judgment or treatment of an individual or group resulting from one’s lack of awareness or ability to effectively regulate activated stereotypes or attitudes. In this way, biased outcomes can result from a set of either implicit (e.g., lacking awareness) or explicit (i.e., lacking motivation to be unbiased) processes.

A fifth pitfall is to assume that awareness of one’s implicit stereotypes and attitudes will eliminate them. Well-designed field studies have directly tested the effectiveness of increasing awareness among actual employees of a large professional services firm about the effects of implicit associations on decision-making (Chang et al., 2019). In this study, employees viewed an hour-long educational video covering either the science of gender bias, biases more generally, or open-communication practices that have nothing to do with gender or bias. Chang and colleagues discovered that learning about implicit biases in general or gender bias more specifically (compared with a control condition), along with strategies for controlling those biases, did in fact lead to increased support for women and acknowledgment that gender bias is a problem, as well as the intention to support inclusion initiatives. Unfortunately, however, these increases in bias awareness and intentions did not translate into changes in behavior. When later given the opportunity to mentor new employees or nominate someone for recognition, people who underwent the online antibias training *were not* more likely to act in supportive ways toward women in their organization. Thus, a fifth pitfall of antibias training is to assume that increasing one’s awareness and understanding of implicit bias is enough to reduce its effects on behavior. Awareness might be a necessary component, but it is far from sufficient.

Finally, a sixth common pitfall is that many training programs focus on educating an individual without considering the broader cultural context in which that individual lives, works, or learns. If biased outcomes result from a process that unfolds over time, we must also acknowledge that the social norms of the surrounding context shape that process (Crandall & Eshleman, 2003). Even if training programs successfully educate individuals about the existence of bias, including those they personally possess, these broader cultural norms and the systems in which they are ensconced are likely to counteract these efforts. Thus, any training program must be deployed in tandem with work by organizational leadership to change policies and practices that reduce structural biases, in part by fostering more inclusive norms.

To sum up, these six critical pitfalls of antibias training programs point to a growing need for a more precise conceptualization of bias that can help scaffold interventions to reduce biased outcomes and foster inclusion. This conceptualization needs to be easy to understand by nonspecialists but grounded in theory and evidence. With that need in mind, we introduce a typology of bias that uses a dual-process approach to identify distinct pathways to biased expression. Although we focus initially on the biased behavior of individuals, we assume that individually held stereotypes and attitudes are perpetuated by and help to perpetuate broader cultural norms and systems of inequality and underrepresentation that must also be addressed within organizations and institutions. We conclude by using our typology to reveal how antibias interventions can target different types of bias by focusing on the distinct pathways of biased behavior. Without differentiating these distinct pathways, antibias interventions will be flawed.

## Biased Outcomes Unfold as a Dynamic and Contextualized Process

### The dual-process approach

The traditional dual-process approach to stereotyping and prejudice asserts that biased outcomes unfold from a process in which the activation of stereotypes and attitudes in individual minds, when unregulated, lead to behavioral reactions that discriminate against others on the basis of their group membership (Devine, 1989; Greenwald & Banaji, 2017). Thus, there are multiple routes by which bias-relevant cognitions in the mind can lead to biased behavior resulting from both automatic and controlled components of mental processing (Kunda & Spencer, 2003; Payne, 2001). Stereotypic beliefs and prejudicial attitudes are first activated automatically (i.e., brought to mind quickly, and often without conscious intent, when one encounters a socially devalued group; Gilbert & Hixon, 1991), but the activation of stereotypes or attitudes does not necessarily lead to biased expression; they can be deliberately controlled or downregulated, often depending on one’s chronically accessible goals, motivations, and intentions, to be egalitarian or situational constraints on enacting biases (Plant & Devine, 1998). Conventional antibias trainings, however, often conflate the existence, measurement, or activation of stereotypes or negative attitudes with evidence for biased outcomes.

One key takeaway from the dual-process literature is that bias is not a static trait in an individual’s mind but rather the outcome of a process that is *dynamic* over time and *embedded* within a social context. For example, social-neuroscience studies have revealed that although the activation of prejudice happens automatically, this response can be perceived as conflicting with one’s egalitarian goals (Amodio et al., 2004), triggering prefrontal downregulation of this initial response (Cunningham et al., 2004). For example, awareness of policies or norms prohibiting the use of racist or sexist language might lead people to inhibit an insensitive question or comment that might have come to mind spontaneously. However, this downregulation is less likely to occur if other environmental cues seem to justify the activated stereotype (Forbes et al., 2012).

A second key takeaway is that bias often results from a *motivated process* (Kunda & Sinclair, 1999). Because stereotypes in particular are heuristics we use to test hypotheses about other people’s actions and intentions, they are strategically activated to help us make sense of our social surroundings (Darley & Gross, 1983). Our motivation to find common ground can lead us to suppress automatically activated stereotypes (Fiske & Neuberg, 1990). In contrast, the motivation to bolster our sense of self, the in-group, or the status quo can justify using biases to shape decisions and behavior that facilitate those goals (Crandall & Eshleman, 2003; Fazio & Towles-Schwen, 1999). For example, although stereotypes and prejudices once activated can fade as individuals become better acquainted, encountering a point of disagreement can lead stereotypes to be reactivated (Kunda & Spencer, 2003). Understanding the role of motivation in bias is central to developing effective interventions to counteract biased behavior. And yet, antibias trainings often overlook the role of one’s motivations to be egalitarian, as noted above.

### A clearer definition of what implicit bias is

The notion that bias can be implicit first emerged when researchers began highlighting the distinction between the automatic activation of learned stereotypes and attitudes and the more controlled process of regulating the expression of these thoughts and feelings to be in line with our goals and values. This distinction between activation and expression set the stage for researchers to develop new measures of implicit stereotypes and attitudes as something distinct from explicit beliefs that can be self-reported on questionnaires (Greenwald & Banaji, 2017). Scholarly work grounded the notion of implicit cognition within our associative networks—integrated webs of concepts that form over time through repeated exposure and learning (Smith & DeCoster, 2000). Research advanced significantly after the emergence of methods for measuring implicit associations, such as the IAT.

Pinpointing exactly what we mean by implicit bias is a challenge. Is implicit bias the negative association that has the potential to be activated automatically? Is it the automatic process by which these associations are activated as a mental state? Or is it the discriminatory behavior that results from the activation of implicit associations? We are not the only ones to point out how imprecision in such terminology can stymie both theoretical and practical advances (e.g., Corneille & Hütter, 2020; De Houwer, 2019).

With the goal of improving the precision of antibias training, we focus on social biases as the outcome of a set of processes by which the activation of group-relevant cognitions (e.g., stereotypes or attitudes) lead to, or influence, one’s behavior toward a member(s) of that group. Implicit bias results from a lack of awareness or ability on the part of an otherwise egalitarian-motivated perceiver to effectively regulate behavior. The same outcome would be labeled explicit, or intentional, bias if the perceiver is unmotivated to counteract how their stereotypes or prejudices affect their behavior. Notably, our focus here is on social biases; the broader literature on cognitive biases falls outside the scope of this article.

To be clear, it is useful to say a few words about what implicit bias is not: It is not necessarily unconscious (Gawronski, 2019). It is also not merely what an implicit measure such as the IAT assesses, because conflating the two obscures the distinction between measurement and construct. Rather, we assume that individuals and cultures vary in the strength of associations they hold toward a given group of people. When these associations are activated in working memory, they have the potential to be expressed in one’s behavior toward a group and its members. Implicit associations and explicit beliefs in the minds of perceivers are the inputs to this process but are unwieldy labels. Thus, within this article, we use the acronym BIASes (beliefs and implicit attitudes and/or stereotypes) to refer to the mental constructs that can lead people to act or react in ways that adversely affect targeted individuals or groups. Although individuals vary in the strength of these BIASes, our focus is on expressions of bias as situational events and not on individuals who are dispositionally biased. Nor are we focused here on the process by which those BIASes are formed in the first place.

## A Typology of Bias

### The antecedents of BIAS expression and regulation

Our bias typology summarizes when and how BIASes are expressed in behavior to better inform antibias training interventions. More concretely, interventions must be mindful of the fact that the expression of BIASes in behavior depends on three particularly key ingredients: perceivers’ underlying motivation to control bias, awareness that control is needed, and the ability to successfully regulate their responses.

#### Motivation

First, the motivation to be unbiased or egalitarian is critical to the control of biased outcomes. Although people can be extrinsically motivated to inhibit their BIASes (i.e., motivated to control their beliefs, implicit attitudes, and stereotypes out of fear of negative consequences if they do not; Plant & Devine, 1998), we focus on times when people feel internally motivated to control their BIASes because they feel this is the appropriate response and/or one consistent with their egalitarian values. Although interventions and proscriptive policies can activate an external motivation, we focus on how interventions might elicit or increase one’s internal motivation to be egalitarian or target social norms that bypass the need for individual motivation.

#### Awareness

Second, awareness refers to an acknowledgment of one’s BIASes and their potential to shape behavior in a given context (Hahn et al., 2014). Indeed, *active* regulation of BIASes requires not only an awareness that the associations exist but also acknowledgment that those BIASes have the potential to affect behavior and do harm in that given moment. Norms in a setting can also modulate people’s awareness of their BIASes.

#### Regulation

Third, regulation refers to a person’s ability and effort in the moment to control their behavior and/or the cognitive processes relative to the demands of a situation. Notably, although stereotyping and prejudice research often focuses on people’s abilities to downregulate a negative belief or attitude that has been activated, when people feel that their BIASes are justified, they might also upregulate their negative response to others (Forscher et al., 2015). Thus, when discussing regulation, we refer to one’s ability to regulate their behavior and whether the type of regulation used is actually effective at reducing harm to others.

**Fig. 1.**
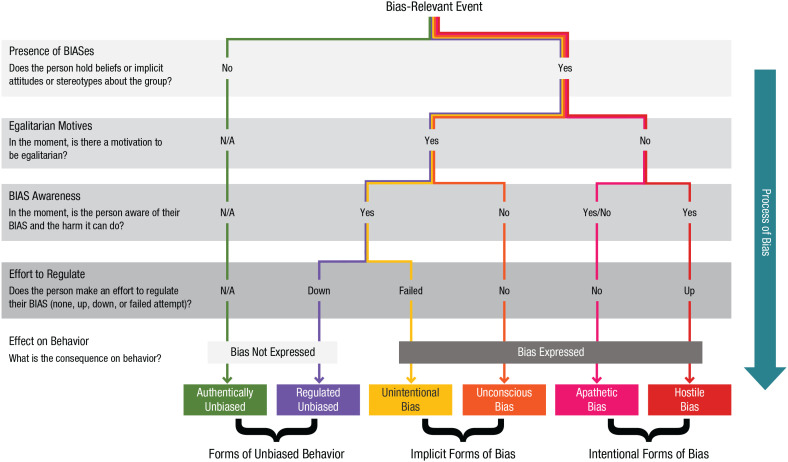
The bias typology: pathways to bias produce different types of bias expression. This figure can be read from top to bottom as a decision tree. Each color represents a different type of bias, each distinguished with a unique label. BIAS = beliefs, implicit attitudes, and/or stereotypes.

In sum, the expression of BIASes is not inevitable, but successful regulation depends on the joint possession of motivation, awareness, and the skills to regulate behavior. The absence of one or more ingredients can lead to distinct pathways for our BIASes to influence behavior in harmful ways (see Fig. 1). Although we articulate these pathways as a typology, we recognize that human psychology does not conform to types. That said, this parsimonious representation has pragmatic value for those interested in translating the basic science of bias into interventions. Finally, our focus on biased outcomes as distinct events that unfold as part of a dynamic and contextualized process means that the presence or absence of a given factor (awareness, motivation, regulation) refers to its presence or absence in that moment. The same individual could in different settings express or control the same BIAS toward others. In addition, as discussed later, norms can stabilize these processes by justifying or policing biased outcomes, representing another critical factor for the implementation and evaluation of antibias training programs.

### Pathways to intentional bias: Motivation or the lack thereof

We begin with the most blatant forms of bias—intentional biases. Expressions of intentional bias come in two forms: *hostile* and *apathetic*. Instances of hostile bias result from a motivation to enact or upregulate the expression of BIASes on behavior (bias by commission), whereas instances of apathetic bias result from a lack of motivation to control or downregulate the expression of BIASes on behavior (bias by omission). These are intentional biases because the outcome aligns with one’s motivations regardless of whether that outcome results from action or inaction.

#### Hostile bias

In our typology, hostile bias results from the intentional upregulation of BIASes on one’s actions. Because hostile bias implies that a person feels justified in holding their attitudes and stereotypes and are motivated to express them, biases of this type are not implicit—they are explicit and deliberate. This kind of bias is often called “old-fashioned prejudice” to contrast it with modern or symbolic forms less directly aimed at their targets (Gaertner & Dovidio, 2000; Sears & Henry, 2005). Research has shown that some people are in fact motivated to express prejudice against out-groups (Forscher et al., 2015).

Self-reported prejudices and stereotypes against many groups (although not all) have been declining over the past several decades. In recent years, people report having less negative beliefs and stereotypes about racial and sexual minorities, but negative attitudes toward those who are overweight have not declined (Charlesworth & Banaji, 2019). These varying changes in attitudes likely reflect different norms that allow people to feel justified not just in having but also in expressing their BIASes about some groups and not others (Crandall & Eshleman, 2003).

Despite these general declining reports of hostile bias, the resurgence of right-wing populism, nationalism, and xenophobia in many countries has emboldened some people to express hostile bias as a badge of honor, wield it as a weapon, or amplify it in the name of free speech (Crandall et al., 2018; Forscher & Kteily, 2020). For example, when a White woman in Central Park called the police to make a false claim that a Black man was threatening her (he had merely asked her to leash her dog in accordance with park rules), she made a conscious decision to mention his race as if that justified her perception of the situation as a threat (Ransom, 2020). Likewise, the proliferation of anti-Asian hate crimes as the COVID-19 pandemic hit North America points to ways in which the physical threat of infection emboldened some to express their racial biases (Gover et al., 2020). And when right-wing supporters of Trump stormed the U.S. capitol building on January 6, 2021, many did so while displaying overtly racist paraphernalia (Simon & Sidner, 2021).

#### Apathetic bias

Apathetic bias occurs when people who are aware of their BIASes are unmotivated to control them. As a result, they make no attempt to monitor the situation or their own behavior or to downregulate their BIASes in the moment. The lack of motivation felt when someone is apathetic to bias can result from different factors. First, apathetic bias can occur when someone is unmotivated to care about or make an effort to regulate a given form of BIAS. For instance, a professor who is privately irritated by a nonbinary student’s pronouns may be successful at using more inclusive language in their large lectures but knowingly misgender the student in a private conversation with a colleague. In this case, they might feel unmotivated to exert the extra effort to regulate their language.

Second, apathetic bias can occur in situations in which other motivations for self-or group interest compete with a person’s chronically held egalitarian motives. This often happens in political contexts in which broader support for egalitarian policies breaks down when those policies affect someone personally. Consider an example in which a family supports the racial integration of schools in principle but then opposes having their own child bussed to another school.

Finally, a third form of apathetic bias reflects uncritical compliance with a prejudiced norm. When people fail to engage in critical self-reflection, they may thoughtlessly reproduce harm stemming from cultural, structural, and systematic prejudice (Salter et al., 2018). For example, apathetic bias might lead instructors to make no attempt to diversify their syllabi, hiring committees to assume that their gender-imbalanced applicant pool is only a pipeline problem, or researchers to adopt practices that exclude or misrepresent more diverse populations. Apathetic bias can be—and quite often is—enacted by people whose chronic egalitarian motives are high. And yet situations can cue other competing motivations, including a basic desire to conserve energy or conform to others’ inaction, or to avoid self-criticism that temporarily reduces the likelihood that a person expends the necessary effort to downregulate the degree that their BIASes bleed into behavior. The harm to targets might feel too small, distant, or abstract to cue a motivation to regulate their behavior in the moment. Nonetheless, apathetic bias contributes to perpetuating norms of biased behavior that causes real harm to targets of those expressions.

### Pathways to implicit bias: failures of awareness and regulation

Implicit bias, we assert, is the expression of discriminatory actions or judgments against a person or group that result from BIASes that the perceiver was either unaware of (i.e., unconscious bias) or unable to effectively regulate (i.e., unintentional bias) in that moment. In our typology, we define implicit bias not by the size or subtlety of the behavior^1^ or the relative impact or harm to a targeted person or group but rather by the absence/failure of awareness or regulation.

#### Unconscious bias

In our typology, unconscious bias refers to discriminatory behavior or judgment that occurs when people are unaware or fail to realize the effect of their BIASes on their behavior in a given situation. This definition, with its focus on awareness of the expression of discriminatory behaviors, is distinct from a popular conception of “unconscious bias” as people lacking awareness of the mental contents of their mind. It is also distinct from the notion that implicit associations reveal hidden prejudice and stereotypes that people are unwilling to admit even to themselves. In fact, people can, to some degree, accurately estimate their implicit associations (Gawronski, 2019; Hahn et al., 2014).

Critically, even if people have insight into the implicit associations in their mind, they might not be aware of how or when those associations affect their behavior in a given context. Their awareness can be influenced by cues in the context, the nature of the specific BIAS, and their own expertise in recognizing the effects of BIASes. For example, a well-meaning hiring manager may explicitly value diversity, but if they also assign value to whether a given candidate seems like a “cultural fit,” their ultimate hiring decisions may reflect a preference for homophily (Rivera, 2012) and, by extension, be biased against minority candidates. The same individual might not exhibit a similar bias against promoting an already successful minority colleague.

A key aspect of unconscious bias is that regardless of whether people have insight into their stereotypes and prejudices, it is harder to be aware in the moment of how those cognitions shape action (Nisbett & Wilson, 1977). Without this awareness, even the most well-intentioned person will exhibit biased behavior. To rectify unconscious bias, people need to become aware not only of their own BIASes but also of the contexts in which BIASes affect behavior.

#### Unintentional bias

Unintentional bias occurs when people who are aware of and motivated to control their BIASes nonetheless fail to successfully regulate their biased behavior. Instances of unintentional bias can be carried out by egalitarian-minded people who have insight into and disdain for their own implicit stereotypes and attitudes and who are quite aware of the contexts in which these associations could bias their actions. Nonetheless, they might lack effective strategies for breaking the link between their implicit associations and behavior.

Unintentional bias can happen when regulatory abilities are insufficient or overwhelmed by the other demands of the context. Consider “foot-in-mouth disease,” in which someone’s biased utterance is accompanied a second later by a wince and apologies. For example, a White supervisor who, in the midst of a group presentation, momentarily confuses the names of her two Asian women employees. Even when immediately corrected, confusing two people of the same race often reflects an out-group homogeneity effect (Ostrom & Sedikides, 1992), the tendency to more easily confuse other-race individuals. In a more relaxed setting, she might have regulated this bias, but regardless of her intention, the consequence of this behavior is that the supervisor’s Asian employees may feel othered or isolated because of their race.

A second type of regulatory failure is when people effectively control the content of what they say but fail to regulate the manner in which they express it. For example, people’s explicit motivation to be nonprejudiced predicts the extent to which they express nonbiased statements, but their implicit racial attitudes are still telegraphed nonverbally (Dovidio et al., 2002). Likewise, a person with implicit antigay attitudes may be unaware that they maintain greater physical distance from a gay coworker relative to their straight colleagues.

Finally, unintentional bias can also occur from using a well-meaning strategy that is unintentionally harmful. For example, White Americans who claim to not “see race” might intend to signal that they believe in racial equality without realizing that this color-blind ideology fails to appreciate the important role that race plays in people’s identity and lived experience (Apfelbaum et al., 2008). Another example is when efforts to express a positive impression convey a stereotyped lens, such as when a White faculty member describes a Black student as articulate, betraying low expectations for minority students. Well-meaning advice also falls under this umbrella, such as when people earnestly ask whether their chronically ill friend is exercising and eating right, conveying an attribution of illness to controllable lifestyle choices.

### Pathways to unbiased behavior: when motivation, awareness, and regulation align

#### Regulated unbiased behavior

Biased expression is not inevitable; people can and often do exhibit regulated unbiased behavior when they successfully downregulate or inhibit the expression of these biases (Fazio & Towles-Schwen, 1999; Plant & Devine, 1998). In fact, regulated unbiased behavior is the realistic goal of antibias training. However, when left to the individual, the successful regulation of BIASes is contingent on people having the awareness, motivation, and ability to deploy effective strategies to counteract their BIASes.

There are many cases in which people can successfully regulate the influence of BIASes on their behavior. For example, those who are internally motivated to respond without sexism are less likely to laugh at or make sexist jokes (Klonis et al., 2005), although they likely hold implicit sexist attitudes or beliefs. Regulated unbiased behavior need include not only the inhibition of BIASes that come to mind but also active efforts toward creating unbiased outcomes. In 2019, Francis S. Collins, the director of the National Institutes of Health, tweeted the following: “Ending the #manel begins at the top. Starting now, I expect a level playing field at public speaking events with a diversity of ideas or I will decline to participate. I challenge other leaders to do the same” (Box, 2019). With an awareness that all-male panels (manels) reflect bias, Collins provides a clear example of regulating his own behavior to be unbiased while creating a norm for others to do the same, a topic we return to below.

#### Authentically unbiased behavior

The idealistic goal of antibias training is to equip people to become authentically unbiased. Unfortunately, this is not likely to be realistic. Authentically unbiased behavior can imply that individuals have no implicit associations that would lead them to disadvantage one group over another. This most commonly occurs when a bias is either not present or is irrelevant in a given context. For instance, a North American with little knowledge of the Indian caste system may not have preexisting attitudes or stereotypes about the Dalit caste who have been historically oppressed in India.

Can people be authentically unbiased when they are exposed to cultural stereotypes or have a strong automatic tendency to positively evaluate the in-group? Given that implicit associations are present by 1 year of age (Pun et al., 2018), we might often assume the presence of implicit stereotypes or attitudes about a variety of social groups. That said, a key question becomes not whether certain people harbor implicit associations but rather how strongly they hold those associations. Some people will have no strong negative out-group associations because of a personal history of positive experiences with or exposure to out-group members who contradict negative stereotypes (Aron et al., 2004; Dasgupta & Asgari, 2004; Pettigrew & Tropp, 2006).

In addition, certain people might not easily develop negative associations of any kind. Livingston and Drwecki (2007) backward-engineered the authentically unbiased person by identifying White college students who exhibited no implicit or explicit racial prejudice (i.e., low on external motivation and high on internal motivation to be unbiased) and were nominated by Black peers to be racially unbiased. Compared with other White students who did not meet these criteria, the authentically unbiased White students were less susceptible to learning negative associations to neutral stimuli and somewhat more likely to learn positive associations. Thus, a general insensitivity to evaluative conditioning might explain who is likely to be authentically unbiased toward a range of different social groups.

Young children may fall into this category. Although childhood represents a critical period for the development of BIASes (Baron, 2015; Bigler & Liben, 2006), there is variability in which social categories children represent, whether they have formed positive or negative associations with those groups, and the relative importance of those intergroup evaluations. For example, children tend to categorize others on the basis of gender and language before race, and positive evaluations of the in-group emerge before negative evaluations of the out-group (Rhodes & Baron, 2019). Such findings suggest that negative out-group BIASes may form only after protracted social learning (Baron, 2015; Pun et al., 2018).

Although we have focused on people without BIASes, it is worth noting that even people who hold such BIASes can behave in an unbiased way if the context never brings those cognitions to mind. For example, race may become less salient if an intergroup competition makes team affiliation more relevant to the context (Kurzban et al., 2001). Thus, a person with BIASes does not *always* engage in biased behavior; a necessary but not sufficient prerequisite is that the context makes such BIASes relevant

#### Summary

Our bias typology specifies how in-the-moment awareness, motivation, and efforts to regulate behavior jointly shape the translation of individuals’ BIASes into behavior within a given context. By visually representing the different pathways of bias expression, we can see the distinction between intentional/explicit forms of bias, unintentional/implicit forms of bias, and situations in which behavior is unbiased. However, to this point, we have described the process by which BIASes in a person’s mind are expressed versus regulated as “slices” of time within a specific context, largely considering individuals as *independent* actors. Contextual factors and social norms also shape these processes through the minds of *interdependent* actors.

## Systemic Bias: From Individual Minds to Cultural Norms

Interventionists often assume that they can change organizational culture by changing the hearts and minds of individuals in that organization. Yet what targets of bias often report is not a series of unrelated biased episodes but episodes that reveal patterns of BIASes embedded in a culture and perpetuated by social norms (Markus & Kitayama, 2010), albeit enacted by a minority of people (Campbell & Brauer, 2020). Thus, efforts to counteract biased behavior at scale hinge on changing the cultural context.

In this section, we expand the traditional dual-process model to include norm-based processes that are dynamically distributed between the minds of interdependent actors. We review the impact that norms can have on attitudes and behavior, introducing the notion of a *distributed dual-process model.* Because bias unfolds within cultural contexts, we explain how the norms of that setting can reinforce and accentuate biases over time, leading to more trait-like modes of thinking about and interacting with others. In addition, we consider the effects of these norms on the ways in which targets interpret and are affected by instances of bias.

### The distributed dual-process model of bias

Norms are potent vehicles for social change. Research from both the lab and the field reveals that norms influence the expression of BIASes (Paluck, 2011; Paluck et al., 2016; Stangor et al., 2001; Sechrist & Stangor, 2001). Norms can shape behavior by changing people’s attitudes (Murrar et al., 2020). But norms can also influence behavior *without* conscious endorsement by those whose behavior they shape (Nolan et al., 2008). That norms can affect behavior independently of attitudes has led some to suggest that changing norms—or at least the *perception* of norms—may be a more viable strategy for cultural change than changing attitudes (Paluck, 2011; Prentice & Paluck, 2020).

We expand the traditional dual-process approach to consider the person in context, in which the process of activating and regulating BIASes is distributed dynamically among the minds of interdependent actors (a distributed dual-process model). Norms are thus affordances in the environment that enable or inhibit the expression of BIASes. For example, Crandall and Eshleman’s (2003) justification-suppression model (JSM) suggests that people express their true prejudicial beliefs about groups when they feel that the norms in the surrounding context justify those prejudicial attitudes (i.e., hostile biases in our typology that people feel motivated to express and even intentionally upregulate). In contrast, when the collective norms support equality and inclusion, people actively suppress their biased beliefs and attitudes (this suppression, if successful, is what we have labeled “regulated unbiased behavior”). As in the JSM, we consider how norms can affect biased expressions. We posit that norms not only shape an individual’s behavior in a top-down, deliberative way but also affect activation, motivation, and regulation efforts.

First, the real or imagined presence of prejudiced others may activate BIASes people neither endorse nor are aware they harbor. Vial et al. (2019) demonstrated that people make biased and discriminatory hiring decisions that go against their own beliefs if they believe their supervisor is prejudiced. Although they may do so out of a fear of punishment, desire to curry favor, or motivation for conformity, it is plausible that the mere presence of prejudiced others could itself increase the likelihood that BIASes are activated. Likewise, the presence of egalitarian others might inhibit the activation of those BIASes.

Second, others in the setting can also cue a motivation to suppress the effects of BIASes on behavior. Consider how the expression of BIASes can change when people are making decisions in diverse groups. Diverse juries produce less racially biased verdicts, in part because White jurors are more motivated to avoid relying on stereotypes as heuristics in favor of a careful review of the evidence (Sommers, 2006). In this context, the presence of diverse others cues people to take into consideration other members’ points of view, motivating them to regulate their BIASes as they attempt to objectively evaluate evidence.

Finally, others in the setting might play an active part in coregulating each other’s biased expressions. For example, Régner et al. (2019) examined whether the average implicit stereotypes held by members of evaluation committees predicted a change over time in the gender balance of hiring decisions for elite science positions. Among committees whose members rejected the notion that women face barriers to success, implicit stereotypes predicted hiring decisions that favored men over women. But among committees whose members explicitly acknowledged that biases might be a problem, implicit stereotypes did not predict more disparate hiring outcomes for women. Committees explicitly concerned about biased selection decisions might have been more likely to deliberately discuss these concerns and work to collectively to set aside and thus regulate their implicit stereotypes.

Admittedly, the examples above suggest rather than provide precise evidence for the mechanisms by which the social context or surrounding norms influence the exact point in the process by which biased outcomes unfold. In reality, multiple mechanisms might be at play and the effect of social norms on these processes might operate in either explicit or implicit ways. However, such findings are consistent with a distributed dual-process model of bias.

This distributed dual-process model can lead to both stability and change in the expression of bias. In the aggregate, one sees stability in biased expression both over time and across individuals who are subjected to those same norms. For example, Payne and colleagues (Payne et al., 2017; Vuletich & Payne, 2019) have suggested that implicit associative measures of prejudice tap into the bias of the surrounding culture as much if not more than individual differences in attitudes. Their research suggests that the network of BIASes manifesting through norms in the culture is more stable than the BIASes measured in individual minds. If this “bias-of-the-crowds” view is accurate, then trying to change the implicit associations or the biased behaviors of individuals is bound to be ineffective without changes to the broader culture. Furthermore, because people tend to self-select into situations with like-minded others (Schmader & Sedikides, 2018), the homogeneity within the cultural context will only reinforce and stabilize patterns of bias expression. As a result, polarization and stabilization of BIASes and their expression becomes even more potent over time.

The broader point is that an individual’s motivation to regulate their BIASes can be influenced by the real or imagined attitudes of others in the social context. The presence of shared beliefs in equality makes it easier to automatically inhibit the activation of BIASes or downregulate them once activated (Moskowitz et al., 1999). There is clearly a need for more research that specifically tests how norms in the surrounding context form and shape activation, motivation, and regulation as distinct components of the pathways to how bias is expressed.

### The target’s dilemma

Our bias typology identifies distinct pathways to biased behavior toward the goal of identifying entry points to disrupt biased behavior. As such, it is more useful as a guide for intervention rather than for characterizing what it is like to be on the receiving end of different types of bias expressions. In an isolated encounter, targets of bias face a dilemma in that they (or other observers) cannot readily distinguish between each type of bias (e.g., unintentional and apathetic bias). That actors’ intentions can be a cipher should be unsurprising given that actors themselves lack introspective insight into the processes that guide their action (Nisbett & Wilson, 1977). In all but the most hostile forms, a given biased expression is not diagnostic of any one process or pathway. For instance, if a White teacher tells a Black student, “Everyone can succeed in this society, if they work hard enough” (Williams, 2020), their utterance could be an intentional jibe reflecting a belief that Black people are lazy (hostile bias), could reflect ignorance (unconscious bias) that espoused meritocracy beliefs disregard systemic issues leading to unequal opportunities, or could stem from a genuine belief (even if misguided) that focusing on hard work is a way to motivate members of disadvantaged groups (unintentional bias).

This attributional ambiguity leaves targets (and other observers) in the difficult position of having to decode an actor’s intentions without reliable indicators of their true motives (Major et al., 2013). This task is more difficult if people’s declared egalitarian motives mask underlying BIASes. And yet the mere uncertainty of ambiguous expressions of bias can create physiological stress—arising from constant vigilance and a lack of control over outcomes (Derks & Scheepers, 2018; Salomon et al., 2015). In this way, the general ambiguity of the pathway to biased behaviors from the perspective of targets adds to their allostatic load, which has implications for weathering and health consequences across the life span (Major & Schmader, 2018; Simons et al., 2018). Implicit and ambiguous expressions of bias can cause equal degrees of harm to targets of bias (Jones et al., 2013), although observers construe implicit bias as less problematic (Daumeyer et al., 2019).

Targets can and do experience harm from implicit bias, but the attribution(s) a target makes can also shape the harm they incur (Major & Dover, 2016). In a single ambiguous encounter, it is adaptive for targets to ascribe negative experiences to another person’s prejudice rather than to themselves (Crocker et al., 1991). However, when biases are recognized as being systemic, attributing one’s treatment to discrimination is more harmful to mental and physical health (Dolezsar et al., 2014; Pascoe & Smart Richman, 2009; Schmitt et al., 2014).

We propose that targets decode ambiguous interactions, in part, by looking to the norms of the current context. When BIASes are assumed to be minimal and genuine egalitarian motives prevalent, targets might ascribe an individual actor’s biased action to implicit rather than intentional bias. Even if the behavior is coded as intentional, broader norms for inclusion might foster resilience to the biased expression coming from a singular prejudiced actor. However, if norms condone the expression of bias, then targets will likely assume that the same action reflects apathetic if not hostile bias. For example, a person who compliments an Asian American for his unaccented English allows a stereotype to inform their impression. If other people in that same context are perceived to be genuinely egalitarian, this one-off encounter might be ascribed to the singular prejudiced actor or potentially interpreted as a verbal slip representing implicit bias, neither of which necessarily herald a persistent threat to inclusion in that context. However, in the context of broader norms of cultural prejudice, the same interaction serves as a powerful reminder that Asian Americans are not fully accepted by the majority group and may reasonably be used as a barometer to forecast their future treatment (Yogeeswaran & Dasgupta, 2010). Thus, norms not only shape how bias is expressed by actors but also how it is perceived by targets, which has implications for the harm that is experienced. Interventions that succeed in creating more inclusive cultural norms will thus not only reduce the likelihood of biased actions but also potentially reduce the experienced harm when expressions of implicit bias do occur.

In sum, although it is difficult to discern which pathway has led to a specific biased event, when an episode of bias is seen as a signal of broader cultural devaluation, either intentional or not, it is more likely to take a toll on targets’ health, well-being, trust, and performance. Our typology provides a theoretically and empirically grounded framework for understanding bias and, importantly, developing effective interventions to disrupt the pathways that lead to biased behaviors. However, it is essential that interventions take into consideration the impacts on targets as well as the larger cultural context in which the interventions take place.

## How the Bias Typology Informs Interventions

Having outlined the BIAS typology and the role of norms in perpetuating systemic biases, we can now identify entry points to successfully mitigate or prevent biased outcomes. Although existing training efforts have a poor track record of changing such outcomes (Dobbin & Kalev, 2016), highlighting pathways to bias can better inform intervention efforts by shifting the focus away from a one-size fits all approach. Toward this end, organizations should begin by identifying the type(s) of biases and cultural norms that are of key concern. By identifying which pathway to target, this framework provides greater precision to inform which type of approach might be best suited to mitigating that bias. We next highlight research that points to efficacious strategies if not bona fide interventions for each approach.

### Changing minds and nudging behavior: achieving authentically unbiased behavior

To achieve authentically unbiased behavior, one can aim to change the degree to which individuals hold BIASes in the first place. The hard way to do this is by changing the implicit associations themselves; an easier strategy is to render them irrelevant to the task at hand.

#### Reducing BIASes: interventions to change implicit associations

Although implicit associations can change, they are stubbornly resistant to intervention efforts. On the one hand, over the past 13 years, race, gender, and sexual orientation stereotypes and attitudes have shifted toward more egalitarian views, whereas negative attitudes toward the elderly and disabled have not (Charlesworth & Banaji, 2019). These data reveal that changes are happening but do not reveal how such associations can be changed or the optimal conditions for engendering that change. Interventions would ideally bring about changes in implicit associations that are qualitative (i.e., negative associations become positive) and enduring (i.e., more than a fleeting occurrence).

Such interventions benefit from understanding when and how implicit associations form. Evidence shows that implicit associations form rapidly, a seemingly mindless outcome when patterns of covariation are detected (Gonzalez et al., 2016; Gregg et al., 2006). Although implicit associations are quick to form, they are slow to change. Meta-analytic reviews (Forscher et al., 2019; Lai et al., 2016) have shown that implicit associations can be temporarily changed by experimental manipulations, but these associations return to preintervention levels hours or days later.

Notably, some strategies hold more promise. In lab studies, exposure to out-group members who contradict group stereotypes and attitudes is relatively more meaningful (Kurdi & Banaji, 2019), and effects are stronger with larger doses of counter-attitudinal information (Rydell et al., 2007). Field studies reveal that positive role models reduce implicit racial bias. For example, medical students who have greater positive contact with a racial out-group show reduced negative implicit attitudes toward that out-group (Van Ryn et al., 2015). Likewise, watching a female avatar communicate a science lesson reduced implicit male = STEM stereotypes among girls and boys (Plant et al., 2009; see also Gonzalez et al., 2017). These studies provide encouraging evidence of successful interventions to change implicit associations.

Institutional policy changes (e.g., public diversity statements) and diversity or bias workshops are unlikely to directly change people’s implicit stereotypes or attitudes. Nonetheless, an organization might incrementally change people’s implicit associations by having a sustained commitment to recruit, reward, and retain positive role models in positions of prestige and leadership (Dasgupta & Asgari, 2004). Stereotypes and attitudes might shift as a result of sustained investment in outreach, hiring, and promotion programs to foster diversity.

The advantage to trying to change implicit associations is that if negative associations can be changed, then perceivers can more easily achieve authentically unbiased behavior or at the very least find it easier to successfully regulate weaker implicit associations. Another advantage of targeting change at this level is that it need not require structural changes to the organization. Creating programs to foster exposure to positive role models can be implemented virtually, globally, and fairly inexpensively. The disadvantage is that implicit associations are difficult to change, reflecting the many years of experience that have shaped them. Thus, interventions might target changes in childhood when associations are more malleable (Baron, 2015; Gonzalez et al., 2021). To achieve lasting change in implicit associations, intensive interventions require sustained effort, which can be plagued by higher rates of attrition. Finally, interventions that do change implicit associations among individuals are highly unlikely to produce organization-wide cultural change in biased expression without other structural changes.

#### Removing temptation: interventions to make implicit associations less relevant

Given the difficulty in changing BIASes, efforts to make BIASes less relevant may hold more promise. In some cases, situations can be structured to avoid activating BIASes in the first place. In one often cited example, orchestras hired more women for top positions after switching to blind auditions in which evaluators did not know the gender of the performer (Goldin & Rouse, 2000). In other contexts, making reviewers unaware of applicants’ gender has led to more women astronomers being granted telescope time (Johnson & Kirk, 2020) and more women coders having their open source code accepted (Terrell et al., 2016).

Granted, it is not always possible to mask applicants’ identities, but other structural changes can still reduce the likelihood that implicit associations are activated. In one study of grant awards (Witteman et al., 2019), the framing of the reviewer instructions had a large effect on the gender ratio of awardees. Women and men were awarded a proportional number of grants when the reviewers were instructed to focus primarily (75% of the total score) on the quality of the ideas in the proposal. However, when reviewers were instructed to focus primarily (75% of the total score) on the leadership record of the principal investigator (for a different type of grant), women were significantly underrepresented in awardees. A simple change in the review criteria dramatically affected gender disparities in outcomes.

A third example of framing effects comes from research on the meritocracy paradox, wherein decision structures that emphasize meritocracy justify biased decision-making. The stereotype that men are more brilliant than women can make men seem like a better fit to positions of intellectual excellence (Bian et al., 2017). Managers asked to assign bonuses to equally productive men and women award higher bonuses to men when the task is framed as rewarding the best performers (Castilla & Benard, 2010). When it is merely framed as a regular performance evaluation, there is no gender gap in bonuses offered. Similar prompts to imagine an “ideal worker” increase racial biases (Brown-Iannuzzi et al., 2013).

The advantage to interventions that structure the situation is to reduce the expression of bias without needing to know much about the variation among individual actors in their motivation or awareness. By locating change in the situation rather than in people, organizations can hopefully sidestep potential backlash against these efforts and foster authentically unbiased outcomes. The disadvantage is that this requires changing all situations in which bias might occur. Such efforts will not capture all manifestations of intentional or implicit bias.

### Counteracting implicit forms of bias

Our typology also provides a foundation for counteracting implicit biases in both its forms. To reduce unconscious biases, trainings should focus on raising people’s awareness of the BIASes they possess, explicitly identifying contexts likely to lead to bias expression and creating plans for the active regulation of behavior. To reduce unintentional biases, interventions should train people how to effectively regulate their BIASes. Several programs of research are highlighting ways to achieve each of these goals.

#### Raising bias awareness: interventions to counteract unconscious bias

Many current antibias training sessions are aimed at increasing people’s awareness of their own BIASes and how they might affect their behavior. Because these sessions are often carried out by for-profit companies or in-house by human resources staff, it is difficult to know how well they reflect the relevant science. Nonetheless, research is beginning to suggest that diversity training can be at least somewhat effective for raising awareness (Bezrukova et al., 2016; Dobbin & Kalev, 2016).

Researchers have investigated different strategies for raising people’s awareness of their own BIASes. Many efforts involve having people learn their score on an IAT, for example, by visiting Project Implicit. A useful aspect of the IAT is its ability to help people become aware of their own implicit stereotypes and attitudes, which they might otherwise be motivated to deny (Morris & Ashburn-Nardo, 2010). Efforts to increase BIAS awareness have the potential to reduce unconscious bias, although the risk of unintentional bias remains.

Another effective strategy is to teach people how to identify implicit bias when it occurs. Moss-Racusin and colleagues (2018) compared people’s reactions to two different kinds of videos on gender bias in STEM: one that featured expert summaries of bias research and a second set in which these gender biases were portrayed in engaging fictional narratives. Both types of videos raised people’s awareness of and accuracy in detecting gender bias. Faculty members were especially persuaded by the expert interviews to increase their support for gender-inclusion efforts, and the narrative videos increased empathy, an emotion that cues bias regulation efforts. The value of expertise in raising awareness extends beyond the lab. San Francisco’s Police Chief credited the social psychologist Jack Glaser in inspiring him to implement policy changes to combat implicit bias in policing (Giuliani-Hoffman, 2020).

Another method of raising awareness is to use interactive games. Shields et al. (2011) created the Workshop Activity for Gender Equity Simulation that allows players to experience firsthand the injustice of having the deck literally stacked against them. People who play the game exhibit greater awareness of gender bias and the problem it creates for gender disparities (Cundiff et al., 2014).

Although raising awareness is a clear and necessary prerequisite to fostering self-regulated unbiased behavior, awareness alone does not change a culture. In addition to the awareness that BIASes exist, people must also be aware of when the BIASes they hold are activated and expressed. One fruitful direction for research is to examine whether awareness interventions are more effective when participants also identify the circumstances under which their BIASes are expressed. People can be taught to regulate (i.e., use top-down control over) their implicit stereotypes and attitudes when encountering an out-group member (Mendoza et al., 2010). That said, a person who is motivated to be egalitarian and who is aware of their BIASes might still fail to regulate them to avoid harm (i.e., unintentional bias). Thus, successful antibias trainings must help people develop skills for effective regulation.

#### Skill development: interventions to counteract unintentional bias

Even when people are intrinsically motivated to control their BIASes, they can still be unsuccessful in regulating them, leading to unintentional bias. In a meta-analysis of 260 samples, awareness-based training was effective at changing people’s attitudes but not changing their behavior (Bezrukova et al., 2016). The most effective trainings combined efforts to increase awareness of BIASes with strategies for changing one’s behavior. Thus, to mitigate unintentional bias, interventions must provide people with effective strategies for disrupting the influence of BIASes on behavior.

One successful example is an intervention by Devine and colleagues that presents bias as a habit to be broken. Their evidence-based training teaches participants to debias themselves using strategies such as (a) replacing stereotypic thoughts with neutral thoughts, (b) imagining people who contradict the stereotype, (c) actively taking the perspective of those from other groups, and (d) increasing one’s opportunities for positive interactions with other groups (Devine et al., 2017). This training has been effective in boosting not just participants’ awareness of implicit bias but also their self-efficacy and reported efforts to counteract their BIASes (Carnes et al., 2015). Two years later, the academic science, engineering, and medicine departments at which the training took place increased their rate of hiring women by 18 percentage points, a marginally significant but preregistered effect in a small sample (Devine et al., 2017). The same intervention led participants to speak out publicly against racism 2 years after the training took place (Forscher et al., 2019). Providing motivated people with tools to increase their awareness and efficacy for regulating BIASes may be a useful strategy for changing people’s behavior.

One disadvantage is that this level of intervention needs to be structured over a longer period of time and integrated into other general training programs, and it might not be easily scalable using multimodal efforts (Bezrukova et al., 2016). Training on “what to do” also assumes that people are motivated to change their practices (Devine et al., 2012). If an organization is plagued by intentional forms of bias, other interventions are called for.

### Change of heart: Mitigating intentional forms of bias

When an organization suffers from intentional forms of bias, efforts should target people’s underlying *motivations* for equality and the *norms* that lead people to feel justified in expressing their BIASes toward others.

A key challenge to moving people from intentional bias to self-regulated forms of unbiased behavior is the difficulty in changing people’s motivations. The typical challenges of changing any behavior are compounded by people’s tendencies to deny one’s biases, favor one’s in-group (Brewer, 1999), and justify the social hierarchy (Jost et al., 2004; Norton & Sommers, 2011; Radke et al., 2020). That these core motivations develop in early childhood (Baron & Banaji, 2009; Newheiser et al., 2014) likely makes them difficult to change. Nevertheless, interventions can use a mix of strategies aimed at reducing threats that trigger these motivated processes, appealing to shared values to be egalitarian, and extolling the benefits that diversity and inclusion bring. In the end, changing norms might be most influential in changing people’s internal motivation to interrogate and counteract their BIASes.

Antibias training can elicit resentment and reactance by those who are not internally motivated to care about diversity and inclusion efforts (Legault et al., 2011). An example of this resentment was on full display when former President Trump banned antibias training for government agencies, declaring it un-American and paradoxically fueling the existence of bias in America. Likewise, recent objections to and prohibitions of teaching critical race theory in the United States have also revealed strong resentment to the idea that systemic bias is real and that discussions of it are both unhealthy and un-American. Although mandatory training programs can be effective in changing attitudes and sometimes behavior (Bezrukova et al., 2016), people have more negative reactions to such programs. Affirmation strategies that encourage people to first reflect on their deeply held values (Steele, 1988) may help mitigate reactance. When people reflect on their core values, they are more open to finding common ground with others, process information in a less biased way, and show a reduction in intergroup prejudice (Badea & Sherman, 2019; Sherman et al., 2017).

Second, concerns for moral fairness predict how one responds to observed bias expression (Goodwin et al., 2020). Thus, tying broadly shared moral concerns, such as fairness, to more focused issues of diversity and inclusion could enhance people’s motivation to support such initiatives. Promoting a social-justice case for diversity efforts (as opposed to a business case) reduces social-identity threat and improves interview performance for women and minorities in business settings (Georgeac et al., 2018). Future work is needed to examine whether a social-justice case for diversity also increases organizational buy-in.

Another way to increase motivation is to sell the benefits of achieving diversity and inclusion rather than the costs of avoiding bias. Diversity can boost creativity, innovation, and more accurate decision-making (Galinsky et al., 2015; Sommers, 2006). Encouraging a growth mindset to improve diversity and value multiculturalism can increase one’s intrinsic motivation (Murphy et al., 2011) and reduce intergroup biases (Plaut et al., 2018; Richeson & Nussbaum, 2004). People who are intrinsically motivated tend to enter intergroup situations with a goal to learn rather than to avoid seeming biased (Plant et al., 2010). Emphasizing the value of multiculturalism alongside merit is especially effective for instilling a sense of inclusion, trust, and acceptance for both minority and majority groups (Gündemir et al., 2017). Finally, boosting feelings of acceptance plays a causal role in increasing people’s internal motivation to be egalitarian (Kunstman et al., 2013).

The advantage of interventions that successfully change motivation is to truly move people away from intentional biases (either hostile or apathetic). But without increasing their awareness of when BIASes might be activated or giving them the skills and ability to downregulate these automatic reactions, they might still express bias. The largest disadvantage in trying to change motivation is that it is quite challenging to counteract people’s motivated reasoning, tap into shared values, and persuade both advantaged and disadvantaged groups that diversity and inclusion can bring benefits for everyone.

### Changing the culture: scaffolding bias control with changes to policies and norms

Interventions designed to retrain implicit associations, increase awareness, impart regulatory skills, and spark motivation help individuals control their own BIASes. However, individual training programs should be implemented within a broader strategy of cultural change (Carter et al., 2020). Organizations can scaffold affordances at three levels where biases can manifest: the individual level, where one’s own BIASes affect judgment and decision-making; the interpersonal level, wherein people can subtly or explicitly coregulate each other’s actions; and the institutional level, when one combats more systemic forms of bias built into programs and policies (Schmader et al., 2020). Thus, we next consider interventions aimed at interpersonal and institutional change.

#### Building bridges: harnessing the power of interpersonal relationships

People’s interpersonal relationships in organizations are critical to their feelings of fit and inclusion (Hall et al., 2019; Schmader & Sedikides, 2018). Thus, antibias training will be more effective if it changes how people interact with one another. For example, training can focus on the most effective ways to confront stereotyping and prejudice (Ashburn-Nardo et al., 2008). Confrontation is effective in reducing biased behavior, although the people confronted initially feel resentful (Czopp et al., 2006).

Confrontation is a form of allyship that is reactive to the presence of some inciting act. But given that marginalized groups often feel a lack of fit and belonging in organizations that have been designed for and by members of the majority (Schmader & Sedikides, 2018), allyship efforts should also be proactive. Training people to take proactive action entails motivating changes in behavior, policies, or practices aimed at increasing a sense of inclusion and respect for those at risk of feeling marginalized (De Souza & Schmader, 2022). Among women in STEM, conversations with male (but not with female) colleagues that signal acceptance predict women’s feelings of inclusion (Hall et al., 2019). Thus, interventions can train members of the advantaged group to engage in active efforts to foster greater inclusion by both reacting to bias when it occurs and proactively signaling respect to those who might feel marginalized.

Ideally, such interventions are successful in changing norms in social networks. In one unique experiment, Paluck (2011) partnered with the Anti-Defamation League to provide antibias peer training to select students in some schools but not others. Students in both schools were later asked to nominate their peers most likely to confront prejudice and were invited to publicly support marriage equality. Results revealed the spread of antibias norms throughout the student networks. Students in the treatment schools recognized that the peer trainers were more likely to confront prejudice, and friends and acquaintances of these peer trainers were more likely to sign a marriage-equality petition. These promising findings suggest that the peer trainers put their training into practice, modeling a norm that spread to other students in their network.

In sum, efforts to change the hearts and minds of individuals are strengthened by distributing efforts for awareness, regulation, or motivation throughout a network. Other people can cue us to regulate our bias and even inspire in us the intrinsic motivation to proactively promote inclusion. These effects can be explicit (e.g., when someone is confronted with their biased behavior) but can also operate more heuristically by harnessing the power of social norms to change behavior outside of direct awareness or intention.

#### Setting the course: communicating norms and values through leadership

Finally, institutions create the scaffolding to help people and their networks counteract their own and each other’s BIASes. Although short-term intervention efforts are unlikely to change or reverse people’s implicit associations or intentional biases, organizational leadership can help foster a sufficiently broad shift in cultural mindsets. Because BIASes not only exist in the minds of individuals but are also distributed across social networks, fostering an environment that reduces their activation and increases regulatory control is a powerful way to curtail the expression of bias. Indeed, a critical mass of actively egalitarian perceivers can motivate each other to be vigilant to and downregulate the expression of bias. How can organizations spark these efforts?

First, organizational messages can signal a shared value for diversity and inclusion. Prodiversity sentiments cue feelings of identity safety and attract more diverse applicants (Chaney et al., 2016; Purdie-Vaughns et al., 2008). The caveat is that mission statements cannot replace accountability. People assume that discrimination is less likely to occur in organizations with salient diversity statements or awards (Kaiser et al., 2013), although minority candidates still face discrimination from ostensibly prodiversity employers (Kang et al., 2016). Thus, prodiversity messaging from leadership needs to be paired with policies and practices that hold people accountable for biased actions, implicit or intentional.

Second, inclusive policies and practices may play a role in fostering inclusive interpersonal norms. In a study of professional engineers (Hall et al., 2018), women working for companies with more gender-inclusive policies and practices reported having more supportive interactions with their male colleagues, which then predicted lower levels of social-identity threat and workplace burnout. Independent of the policies themselves, others’ perceived support of these policies further predicted women’s (but not men’s) organizational commitment (Hall et al., 2021). Thus, as long as there is broad support for having these policies in place, inclusive policies might shape inclusive social norms.

This prior point suggests that organizations should assess, track, and communicate changes in the culture of an organization over time. People tend to underestimate how much the advantaged majority supports inclusion initiatives (De Souza & Schmader, 2022), thus communicating that these beliefs can correct this pluralistic ignorance. In fact, changing people’s perceptions of the normative support for diversity and inclusion increases marginalized students’ experience of peer respect, sense of belonging, and an inclusive climate, as well as then benefiting their health and academic performance (Murrar et al., 2020). These effects of communicating normative beliefs (implemented easily via shared videos) were significantly more beneficial than providing educational information about bias.

The greatest benefit to changing norms is that it does not require buy-in from everyone in an organization. Social groups tend to rapidly adopt a new or minority behavior once uptake exceeds 25% (Centola et al., 2018). This suggests that organizations can focus on getting norm adoption over this critical threshold. Thus, even if interventions are initially effective only with those who are highly motivated, many bystanders will eventually sign on and help to regulate if not reform those with more entrenched and intentional biases.

The clear advantage to changing broader cultural norms is to create long-term cultural shifts. Even if norms lead some people to initially change behavior as a result of external accountability efforts, these patterns of behavioral change might become internalized over time as intrinsically motivated actions that are consistent with core values. The challenge is that durable change at this scale can require strategic planning and a commitment of resources to track metrics over time and adjust one’s approach as needed.

## Conclusion

There is a high demand for theoretically derived and evidence-based interventions aimed at fostering equity, diversity, and inclusion. Those developing and facilitating antibias trainings face an essential challenge in truly increasing people’s awareness, changing negative attitudes and stereotypes, and fostering antibias and inclusive behavior. This challenge is exacerbated by confusion (even among scientists) over what exactly implicit bias is. We take the position that biased intergroup outcomes unfold as a process by which BIASes—in the minds of individuals as well as distributed across their social networks—lead to discriminatory behavior toward others. BIASes can be cued automatically or acted on intentionally, but they can also be downregulated or set aside if one has the awareness, motivation, and ability to do so. These mental processes of the individual are supported by the social norms in the context. Although individuals can vary in the BIASes they hold, situations play a key role in cuing or counteracting people’s behavior.

Drawing on insights from 3 decades of social-psychological research, we have provided a typology of bias that identifies different pathways by which social biases unfold. People exhibit intentional biases when they are aware of their stereotypes and prejudices but either have little motivation to control them (apathetic bias) or are motivated to express them (hostile bias). People exhibit implicit biases when, despite any motivations to be egalitarian, they are either unaware of the need to counteract their own prejudices or stereotypes (unconscious bias) or engage in ineffective strategies to regulate them (unintentional bias). Interventions aimed at educating people about the nature of bias are unlikely to make them authentically unbiased but can have the more modest goal of helping people successfully regulate their biased behavior. Complicating efforts to reduce bias through self-regulation is that surrounding social norms can support, justify, and dynamically regulate the mental processes of individuals. Indeed, understanding how this occurs is a key insight missing in many intervention efforts.

Our typology can be used to effectively target interventions at the problem pathway for a given context. Organizations need to first identify the prominent types of bias occurring in that setting and choose interventions aimed at changing social norms, fostering inclusive interactions, increasing personal motivation for antibias efforts, increasing awareness of when biases are expressed, and/or training people to counteract their own or others’ biases. Training aimed at one process (successful regulation) will be ineffective if the problem lies in another pathway (a lack of motivation; broader norms against equity, diversity, and inclusion efforts).

Our closing note is that, in implementing any interventions, it is crucial to consider that those who experience bias face the predicament of attributional ambiguity, often making it impossible to discern whether a given outcome results from one pathway or another. Broader cultural norms for inclusion are likely to inform targeted group members’ attributions for—and resilience to—encounters with biased behavior, which has implications for their health and well-being. The most effective interventions create partnerships across identity lines, when a critical mass of people within an organization or community work together toward the shared goal of creating an inclusive culture that reduces the harm that bias can cause and fosters well-being for all.

## Transparency

*Action Editor:* Laura A. King

*Editor:* Laura A. King

## Notes

1. Less-than-blatant bias expressions are sometimes labeled “microaggressions,” originally defined as “brief, everyday exchanges that send denigrating messages to people of color because they belong to a racial minority group” (Sue et al., 2007, p. 273). We agree that such everyday bias is harmful to targets, but we also agree that the microaggression construct is “excessively fuzzy,” making it difficult to examine scientifically (Lilienfeld, 2017). For example, the lack of clarity over whether intentionality is a defining feature of all or some forms of microaggressions makes it difficult for us to place the term within the typology we have outlined.

## References

[bibr1-17456916211057565] Amodio D. M. Harmon-Jones E. Devine P. G. Curtin J. J. Hartley S. L. Covert A. E. (2004). Neural signals for the detection of unintentional race bias. Psychological Science, 15(2), 88–93. 10.1111/j.0963-7214.2004.01502003.x 14738514

[bibr2-17456916211057565] Apfelbaum E. P. Sommers S. R. Norton M. I. (2008). Seeing race and seeming racist? Evaluating strategic colorblindness in social interaction. Journal of Personality and Social Psychology, 95(4), 918–932. 10.1037/a0011990 18808268

[bibr3-17456916211057565] Aron A. McLaughlin-Volpe T. Mashek D. Lewandowski G. Wright S. C. Aron E. N. (2004). Including others in the self. European Review of Social Psychology, 15(1), 101–132. 10.1080/10463280440000008

[bibr4-17456916211057565] Ashburn-Nardo L. Morris K. A. Goodwin S. A. (2008). The confronting prejudiced responses (CPR) model: Applying CPR in organizations. Academy of Management Learning & Education, 7(3), 332–342. 10.5465/amle.2008.34251671

[bibr5-17456916211057565] Badea C. Sherman D. K. (2019). Self-affirmation and prejudice reduction: When and why? Current Directions in Psychological Science, 28(1), 40–46. 10.1177/0963721418807705

[bibr6-17456916211057565] Baron A. S. (2015). Constraints on the development of implicit intergroup attitudes. Child Development Perspectives, 9(1), 50–54. 10.1111/cdep.12105

[bibr7-17456916211057565] Baron A. S. Banaji M. (2009). Evidence of system justification in young children. Social and Personality Psychology Compass, 3(6), 918–926. 10.1111/j.1751-9004.2009.00214.x

[bibr8-17456916211057565] Bezrukova K. Spell C. S. Perry J. L. Jehn K. A. (2016). A meta-analytical integration of over 40 years of research on diversity training evaluation. Psychological Bulletin, 142(11), 1227–1274. 10.1037/bul0000067 27618543

[bibr9-17456916211057565] Bian L. Leslie S. J. Cimpian A. (2017). Gender stereotypes about intellectual ability emerge early and influence children’s interests. Science, 355(6323), 389–391. 10.1126/science.aah6524 28126816

[bibr10-17456916211057565] Bigler R. S. Liben L. S. (2006). A developmental intergroup theory of social stereotypes and prejudice. In Kail R. (Ed.), Advances in child development and behavior (Vol. 34, pp. 39–89). Academic Press. 10.1016/s0065-2407(06)80004-2 17120802

[bibr11-17456916211057565] Box O. (2019, June 14). It’s time to cancel the “manel”—All-male panels don’t just lack diversity: They’re actively counter-productive to diversifying STEM communities. Massive Science. https://massivesci.com/notes/nih-francis-collins-manels-diversity-panels

[bibr12-17456916211057565] Brewer M. B. (1999). The psychology of prejudice: Ingroup love and outgroup hate? Journal of Social Issues, 55(3), 429–444. 10.1111/0022-4537.00126

[bibr13-17456916211057565] Brown-Iannuzzi J. L. Payne B. K. Trawalter S. (2013). Narrow imaginations: How imagining ideal employees can increase racial bias. Group Processes & Intergroup Relations, 16(6), 661–670. 10.1177/1368430212467477

[bibr14-17456916211057565] Campbell M. R. Brauer M. (2020). Is discrimination widespread? Testing assumptions about bias on a university campus. Journal of Experimental Psychology: General. 10.1037/xge0000983 33048568

[bibr15-17456916211057565] Carnes M. Devine P. G. Baier Manwell L. Byars-Winston A. Fine E. Ford C. E. Forscher P. Isaac C. Kaatz A. Magua W. Palta M. Sheridan J. (2015). Effect of an intervention to break the gender bias habit for faculty at one institution: A cluster randomized, controlled trial. Academic Medicine, 90(2), 221–230. 10.1097/acm.0000000000000552 25374039 PMC4310758

[bibr16-17456916211057565] Carter E. R. Onyeador I. N. Lewis N. A. (2020). Developing & delivering effective anti-bias training: Challenges & recommendations. Behavioral Science & Policy, 6(1), 57–70. 10.1353/bsp.2020.0005

[bibr17-17456916211057565] Castilla E. J. Benard S. (2010). The paradox of meritocracy in organizations. Administrative Science Quarterly, 55(4), 543–676. 10.2189/asqu.2010.55.4.543

[bibr18-17456916211057565] Centola D. Becker J. Brackbill D. Baronchelli A. (2018). Experimental evidence for tipping points in social convention. Science, 360(6393), 1116–1119. 10.1126/science.aas8827 29880688

[bibr19-17456916211057565] Chaney K. E. Sanchez D. T. Remedios J. D. (2016). Organizational identity safety cue transfers. Personality and Social Psychology Bulletin, 42, 1564–1576. 10.1177/0146167216665096 30208783

[bibr20-17456916211057565] Chang E. H. Milkman K. L. Gromet D. M. Rebele R. W. Massey C. Duckworth A. L. Grant A. M. (2019). The mixed effects of online diversity training. Proceedings of the National Academy of Sciences, USA, 116(16), 7778–7783. 10.1073/pnas.1816076116 PMC6475398 30936313

[bibr21-17456916211057565] Charlesworth T. E. S. Banaji M. R. (2019). Patterns of implicit and explicit attitudes: I. Long-term change and stability from 2007 to 2016. Psychological Science, 30(2), 174–192. 10.1177/0956797618813087 30605364

[bibr22-17456916211057565] Corneille O. Hütter M. (2020). Implicit? What do you mean? A comprehensive review of the delusive implicitness construct in attitude research. Personality and Social Psychology Review, 24(3), 212–232. 10.1177/1088868320911325 32193993

[bibr23-17456916211057565] Crandall C. S. Eshleman A. (2003). A justification-suppression model of the expression and experience of prejudice. Psychological Bulletin, 129(3), 414–446. 10.1037/0033-2909.129.3.414 12784937

[bibr24-17456916211057565] Crandall C. S. Miller J. M. White M. H. (2018). Changing norms following the 2016 US presidential election: The Trump effect on prejudice. Social Psychological and Personality Science, 9(2), 186–192. 10.1177/1948550617750735

[bibr25-17456916211057565] Crocker J. Voelkl K. Testa M. Major B. (1991). Social stigma: The affective consequences of attributional ambiguity. Journal of Personality and Social Psychology, 60(2), 218–228. 10.1037/0022-3514.60.2.218 8421252

[bibr26-17456916211057565] Cundiff J. L. Zawadzki M. J. Danube C. L. Shields S. A. (2014). Using experiential learning to increase the recognition of everyday sexism as harmful: The WAGES intervention. Journal of Social Issues, 70(4), 703–721. 10.1111/josi.12087

[bibr27-17456916211057565] Cunningham W. A. Johnson M. K. Raye C. L. Gatenby J. C. Gore J. C. Banaji M. R. (2004). Separable neural components in the processing of Black and White faces. Psychological Science, 15(12), 806–813. 10.1111/j.0956-7976.2004.00760.x 15563325

[bibr28-17456916211057565] Czopp A. M. Monteith M. J. Mark A. Y. (2006). Standing up for a change: Reducing bias through interpersonal confrontation. Journal of Personality and Social Psychology, 90(5), 784–803. 10.1037/0022-3514.90.5.784 16737373

[bibr29-17456916211057565] Darley J. M. Gross P. H. (1983). A hypothesis-confirming bias in labeling effects. Journal of Personality and Social Psychology, 44(1), 20–33. 10.1037/0022-3514.44.1.20

[bibr30-17456916211057565] Dasgupta N. Asgari S. (2004). Seeing is believing: Exposure to counterstereotypic women leaders and its effect on the malleability of automatic gender stereotyping. Journal of Experimental Social Psychology, 40(5), 642–658.

[bibr31-17456916211057565] Daumeyer N. M. Onyeador I. N. Brown X. Richeson J. A. (2019). Consequences of attributing discrimination to implicit vs. explicit bias. Journal of Experimental Social Psychology, 84, Article 103812. 10.1016/j.jesp.2019.04.010

[bibr32-17456916211057565] De Houwer J. (2019). Implicit bias is behavior: A functional-cognitive perspective on implicit bias. Perspectives on Psychological Science, 14(5), 835–840. 10.1177/1745691619855638 31374177

[bibr33-17456916211057565] Derks B. Scheepers D. (2018). Neural and cardiovascular pathways from stigma to suboptimal health. In Major B. Dovidio J. F. Link B. G. (Eds.), Oxford library of psychology. The Oxford handbook of stigma, discrimination, and health (pp. 241–264). Oxford University Press.

[bibr34-17456916211057565] De Souza L. Schmader T. (2022). The misjudgment of men: Does pluralistic ignorance inhibit allyship? Journal of Personality and Social Psychology, 122(2), 265–285. 10.1037/pspi0000362 33871267

[bibr35-17456916211057565] Devine P. G. (1989). Stereotypes and prejudice: Their automatic and controlled components. Journal of Personality and Social Psychology, 56(1), 5–18. 10.1037/0022-3514.56.1.5

[bibr36-17456916211057565] Devine P. G. Forscher P. S. Austin A. J. Cox W. T. L. (2012). Long-term reduction in implicit race bias: A prejudice habit-breaking intervention. Journal of Experimental Social Psychology, 48(6), 1267–1278. 10.1016/j.jesp.2012.06.003 23524616 PMC3603687

[bibr37-17456916211057565] Devine P. G. Forscher P. S. Cox W. T. L. Kaatz A. Sheridan J. Carnes M. (2017). A gender bias habit-breaking intervention led to increased hiring of female faculty in STEMM departments. Journal of Experimental Social Psychology, 73, 211–215. 10.1016/j.jesp.2017.07.002 29249837 PMC5729935

[bibr38-17456916211057565] Dobbin F. Kalev A. (2016). Why diversity programs fail. Harvard Business Review, 94(7), 14.

[bibr39-17456916211057565] Dolezsar C. M. McGrath J. J. Herzig A. J. M. Miller S. B. (2014). Perceived racial discrimination and hypertension: A comprehensive systematic review. Health Psychology, 33(1), 20–34. 10.1037/a0033718 24417692 PMC5756074

[bibr40-17456916211057565] Dovidio J. F. Kawakami K. Gaertner S. L. (2002). Implicit and explicit prejudice and interracial interaction. Journal of Personality and Social Psychology, 82(1), 62–68. 10.1037/0022-3514.82.1.62 11811635

[bibr41-17456916211057565] Emerson J. (2017, April 28). Don’t give up on unconscious bias training—Make it better. Harvard Business Review. https://hbr.org/2017/04/dont-give-up-on-unconscious-bias-training-make-it-better

[bibr42-17456916211057565] Exec. Order No. 13,950, 3 C.F.R. 433 (2020). https://www.govinfo.gov/content/pkg/CFR-2021-title3-vol1/pdf/CFR-2021-title3-vol1-eo13950.pdf

[bibr43-17456916211057565] Fazio R. H. Towles-Schwen T. (1999). The MODE model of attitude-behavior processes. In Chaiken S. Trope Y. (Eds.), Dual-process theories in social psychology (pp. 97–116). Guilford Press.

[bibr44-17456916211057565] Fiske S. T. Neuberg S. L. (1990). A continuum of impression formation, from category-based to individuating processes: Influences of information and motivation on attention and interpretation. In Zanna M. P. (Ed.), Advances in experimental social psychology (Vol. 23, pp. 1–74). Academic Press.

[bibr45-17456916211057565] Forbes C. E. Cox C. L. Schmader T. Ryan L. (2012). Negative stereotype activation alters interaction between neural correlates of arousal, inhibition and cognitive control. Social Cognitive and Affective Neuroscience, 7(7), 771–781. 10.1093/scan/nsr052 21954239 PMC3475352

[bibr46-17456916211057565] Forscher P. S. Cox W. T. L. Graetz N. Devine P. G. (2015). The motivation to express prejudice. Journal of Personality and Social Psychology, 109(5), 791–812. 10.1037/pspi0000030 26479365 PMC4616257

[bibr47-17456916211057565] Forscher P. S. Kteily N. S. (2020). A psychological profile of the alt-right. Perspectives on Psychological Science, 15(1), 90–116. 10.1177/1745691619868208 31747343 PMC6980479

[bibr48-17456916211057565] Forscher P. S. Lai C. K. Axt J. R. Ebersole C. R. Herman M. Devine P. G. Nosek B. A. (2019). A meta-analysis of procedures to change implicit measures. Journal of Personality and Social Psychology, 117(13), 522–559. 10.1037/pspa0000160 31192631 PMC6687518

[bibr49-17456916211057565] Gaertner S. L. Dovidio J. F. (2000). The aversive form of racism. In Stangor C. (Ed.), Stereotypes and prejudice: Essential readings (pp. 289–304). Psychology Press.

[bibr50-17456916211057565] Galinsky A. D. Todd A. R. Homan A. C. Phillips K. W. Apfelbaum E. P. Sasaki S. J. Richeson J. A. Olayon J. B. Maddux W. W. (2015). Maximizing the gains and minimizing the pains of diversity: A policy perspective. Perspectives on Psychological Science, 10(6), 742–748. 10.1177/1745691615598513 26581729

[bibr51-17456916211057565] Gawronski B. (2019). Six lessons for a cogent science of implicit bias and its criticism. Perspectives on Psychological Science, 14(4), 574–595. 10.1177/1745691619826015 31181174

[bibr52-17456916211057565] Georgeac O. Rattan A. Dobbin F. Akinola M. Mayer D. Ruttan R. L. Williams J. (2018). Business or fairness case for social issues? Influencing stakeholders in organizations. Academy of Management Proceedings, 18(1). 10.5465/AMBPP.2018.14510symposium

[bibr53-17456916211057565] Gilbert D. T. Hixon J. G. (1991). The trouble of thinking: Activation and application of stereotypic beliefs. Journal of Personality and Social Psychology, 60(4), 509–517. 10.1037/0022-3514.60.4.509

[bibr54-17456916211057565] Giuliani-Hoffman F. (2020, July 20). San Francisco Police Department will stop releasing most mug shots to combat racial bias. CNN. https://www.cnn.com/2020/07/02/us/san-francisco-police-mug-shot-release-trnd/index.html

[bibr55-17456916211057565] Goldin C. Rouse C. (2000). Orchestrating impartiality: The impact of “blind” auditions on female musicians. American Economic Review, 90(4), 715–741. 10.1257/aer.90.4.715

[bibr56-17456916211057565] Gonzalez A. M. Dunlop W. L. Baron A. S. (2016). Malleability of implicit associations across development. Developmental Science, 20(6), Article e12481. 10.1111/desc.12481 27785857

[bibr57-17456916211057565] Gonzalez A. M. Steele J. R. Baron A. S. (2017). Reducing children’s implicit racial bias through exposure to positive out-group exemplars. Child Development, 88(1), 123–130. 10.1111/cdev.12582 27392212

[bibr58-17456916211057565] Gonzalez A. M. Steele J. R. Chan E. F. Lim S. A. Baron A. S. (2021). Developmental differences in the malleability of implicit racial bias following exposure to counterstereotypical exemplars. Developmental Psychology, 57(1), 102–113. 10.1037/dev0001128 33252922

[bibr59-17456916211057565] Goodwin R. Graham J. Diekmann K. A. (2020). Good intentions aren’t good enough: Moral courage in opposing sexual harassment. Journal of Experimental Social Psychology, 86, Article 103894. 10.1016/j.jesp.2019.103894

[bibr60-17456916211057565] Gover A. R. Harper S. B. Langton L. (2020). Anti-Asian hate crime during the COVID-19 pandemic: Exploring the reproduction of inequality. American Journal of Criminal Justice, 45(4). 10.1007/s12103-020-09545-1 PMC7364747 32837171

[bibr61-17456916211057565] Green T. L. Hagiwara N. (2020, August 28). The problem with implicit bias training. Scientific American. https://www.scientificamerican.com/article/the-problem-with-implicit-bias-training

[bibr62-17456916211057565] Greenwald A. G. Banaji M. R. (2017). The implicit revolution: Reconceiving the relation between conscious and unconscious. American Psychologist, 72(9), 861–871. 10.1037/amp0000238 29283625

[bibr63-17456916211057565] Greenwald A. G. McGhee D. E. Schwartz J. L. K. (1998). Measuring individual differences in implicit cognition: The implicit association test. Journal of Personality and Social Psychology, 74(6), 1464–1480. 10.1037//0022-3514.74.6.1464 9654756

[bibr64-17456916211057565] Greenwald A. G. Poehlman T. A. Uhlmann E. L. Banaji M. R. (2009). Understanding and using the Implicit Association Test: III. Meta-analysis of predictive validity. Journal of Personality and Social Psychology, 97(1), 17–41. 10.1037/a0015575 19586237

[bibr65-17456916211057565] Gregg A. P. Seibt B. Banaji M. R. (2006). Easier done than undone: Asymmetry in the malleability of implicit preferences. Journal of Personality and Social Psychology, 90(1), 1–20. 10.1037/0022-3514.90.1.1 16448307

[bibr66-17456916211057565] Gündemir S. Dovidio J. F. Homan A. C. De Dreu C. K. W. (2017). The impact of organizational diversity policies on minority employees’ leadership self-perceptions and goals. Journal of Leadership & Organizational Studies, 24(2), 172–188. 10.1177/1548051816662615

[bibr67-17456916211057565] Hahn A. Judd C. M. Hirsh H. K. Blair I. V. (2014). Awareness of implicit attitudes. Journal of Experimental Psychology: General, 143(3), 1369–1392. 10.1037/a0035028 24294868 PMC4038711

[bibr68-17456916211057565] Hall W. Schmader T. Aday A. Croft E. (2019). Decoding the dynamics of social identity threat in the workplace: A within-person analysis of women’s and men’s interactions in STEM. Social Psychological and Personality Science, 10(4), 542–552. 10.1177/1948550618772582

[bibr69-17456916211057565] Hall W. Schmader T. Aday A. Inness M. Croft E. (2018). Climate control: Cultural predictors of social identity threat for women in engineering. Journal of Personality and Social Psychology, 115, 446–467. 10.1037/pspi0000137 30047760

[bibr70-17456916211057565] Hall W. Schmader T. Inness M. Croft E. (2021). Climate change: An increase in norms for inclusion predicts greater fit and commitment for women in STEM. Group Processes and Intergroup Relations. Advance online publication. 10.1177/13684302211035438

[bibr71-17456916211057565] Johnson S. K. Kirk J. F. (2020). Dual-anonymization yields promising results for reducing gender bias: A naturalistic field experiment of applications for Hubble space telescope time. Publications of the Astronomical Society of the Pacific, 132(1009), Article 034503. 10.1088/1538-3873/ab6ce0

[bibr72-17456916211057565] Jones K. P. King E. B. Nelson J. Geller D. S. Bowes-Sperry L. (2013). Beyond the business case: An ethical perspective of diversity training. Human Resource Management, 52(1), 55–74. 10.1002/hrm.21517

[bibr73-17456916211057565] Jost J. T. Banaji M. R. Nosek B. A. (2004). A decade of system justification theory: Accumulated evidence of conscious and unconscious bolstering of the status quo. Political Psychology, 25(6), 881–919. 10.1111/j.1467-9221.2004.00402.x

[bibr74-17456916211057565] Kaiser C. R. Major B. Jurcevic I. Dover T. L. Brady L. M. Shapiro J. R. (2013). Presumed fair: Ironic effects of organizational diversity structures. Journal of Personality and Social Psychology, 104(3), 504–519. 10.1037/a0030838 23163748

[bibr75-17456916211057565] Kang S. K. DeCelles K. A. Tilcsik A. Jun S. (2016). Whitened résumés: Race and self-presentation in the labor market. Administrative Science Quarterly, 61(3), 469–502. 10.1177/0001839216639577

[bibr76-17456916211057565] Kirkland R. Bohnet I. (2017, April 7). Focusing on what works for workplace diversity. McKinsey & Company. https://www.mckinsey.com/featured-insights/gender-equality/focusing-on-what-works-for-workplace-diversity

[bibr77-17456916211057565] Klonis S. C. Plant E. A. Devine P. G. (2005). Internal and external motivation to respond without sexism. Personality and Social Psychology Bulletin, 31(9), 1237–1249. 10.1177/0146167205275304 16055643

[bibr78-17456916211057565] Kunda Z. Sinclair L. (1999). Motivated reasoning with stereotypes: Activation, application, and inhibition. Psychological Inquiry, 10(1), 12–22. 10.1207/s15327965pli1001_2

[bibr79-17456916211057565] Kunda Z. Spencer S. J. (2003). When do stereotypes come to mind and when do they color judgment? A goal-based theoretical framework for stereotype activation and application. Psychological Bulletin, 129(4), 522–544. 10.1037/0033-2909.129.4.522 12848219

[bibr80-17456916211057565] Kunstman J. W. Plant E. A. Zielaskowski K. LaCosse J. (2013). Feeling in with the outgroup: Outgroup acceptance and the internalization of the motivation to respond without prejudice. Journal of Personality and Social Psychology, 105(3), 443–457. 10.1037/a0033082 23750814

[bibr81-17456916211057565] Kurdi B. Banaji M. R. (2019). Attitude change via repeated evaluative pairings versus evaluative statements: Shared and unique features. Journal of Personality and Social Psychology, 116(5), 681–703. 10.1037/pspa0000151 30829506

[bibr82-17456916211057565] Kurzban R. Tooby J. Cosmides L. (2001). Can race be erased? Coalitional computation and social categorization. Proceedings of the National Academy of Sciences, USA, 98(26), 15387–15392. 10.1073/pnas.251541498 PMC65039 11742078

[bibr83-17456916211057565] Lai C. K. Marini M. Lehr S. A. Cerruti C. Shin J.-E. L. Joy-Gaba J. A. Ho A. K. Teachman B. A. Wojcik S. P. Koleva S. P. Frazier R. S. Heiphetz L. Chen E. E. Turner R. N. Haidt J. Kesebir S. Hawkins C. B. Schaefer H. S. Rubichi S. . . . Nosek B. A. (2014). Reducing implicit racial preferences: I. A comparative investigation of 17 interventions. Journal of Experimental Psychology: General, 143(4), 1765–1785. 10.1037/a0036260 24661055

[bibr84-17456916211057565] Lai C. K. Skinner A. L. Cooley E. Murrar S. Brauer M. Devos T. Calanchini J. Xiao Y. J. Pedram C. Marshburn C. K. Simon S. Blanchar J. C. Joy-Gaba J. A. Conway J. Redford L. Klein R. A. Roussos G. Schellhaas F. M. H. Burns M. . . . Nosek B. A. (2016). Reducing implicit racial preferences: II. Intervention effectiveness across time. Journal of Experimental Psychology: General, 145(8), 1001–1016. 10.1037/xge0000179 27454041

[bibr85-17456916211057565] Legault L. Gutsell J. N. Inzlicht M. (2011). Ironic effects of antiprejudice messages: How motivational interventions can reduce (but also increase) prejudice. Psychological Science, 22(12), 1472–1477. 10.1177/0956797611427918 22123778

[bibr86-17456916211057565] Lilienfeld S. O. (2017). Microaggressions: Strong claims, inadequate evidence. Perspectives on Psychological Science, 12(1), 138–169. 10.1177/1745691616659391 28073337

[bibr87-17456916211057565] Livingston R. W. Drwecki B. B. (2007). Why are some individuals not racially biased? Susceptibility to affective conditioning predicts nonprejudice toward Blacks. Psychological Science, 18(9), 816–823. 10.1111/j.1467-9280.2007.01985.x 17760779

[bibr88-17456916211057565] Major B. Dover T. L. (2016). Attributions to discrimination: Antecedents and consequences. In Nelson T. D. (Ed.), Handbook of prejudice, stereotyping, and discrimination (pp. 213–239). Psychology Press.

[bibr89-17456916211057565] Major B. Sawyer P. J. Kunstman J. W. (2013). Minority perceptions of Whites’ motives for responding without prejudice: The perceived internal and external motivation to avoid prejudice scales. Personality and Social Psychology Bulletin, 39(3), 401–414. 10.1177/0146167213475367 23376889

[bibr90-17456916211057565] Major B. Schmader T. (2018). Stigma, social identity threat, and health. In Major B. Dovidio J. F. Link B. G. (Eds.), The Oxford handbook of stigma, discrimination, and health (pp. 85–103). Oxford University Press.

[bibr91-17456916211057565] Markus H. R. Kitayama S. (2010). Cultures and selves: A cycle of mutual constitution. Perspectives on Psychological Science, 5(4), 420–430. 10.1177/1745691610375557 26162188

[bibr92-17456916211057565] Mendoza S. A. Gollwitzer P. M. Amodio D. M. (2010). Reducing the expression of implicit stereotypes: Reflexive control through implementation intentions. Personality and Social Psychology Bulletin, 36(4), 512–523. 10.1177/0146167210362789 20363905

[bibr93-17456916211057565] Morris K. A. Ashburn-Nardo L. (2010). The Implicit Association Test as a class assignment: Student affective and attitudinal reactions. Teaching of Psychology, 37(1), 63–68. 10.1080/00986280903426019

[bibr94-17456916211057565] Moskowitz G. B. Gollwitzer P. M. Wasel W. Schaal B. (1999). Preconscious control of stereotype activation through chronic egalitarian goals. Journal of Personality and Social Psychology, 77(1), 167–184. 10.1037/0022-3514.77.1.167

[bibr95-17456916211057565] Moss-Racusin C. A. Pietri E. S. Hennes E. P. Dovidio J. F. Brescoll V. L. Roussos G. Handelsman J. (2018). Reducing STEM gender bias with VIDS (video interventions for diversity in STEM). Journal of Experimental Psychology: Applied, 24(2), 236–260. 10.1037/xap0000144 29733620

[bibr96-17456916211057565] Murphy M. C. Richeson J. A. Mo lden D. C. (2011). Leveraging motivational mindsets to foster positive interracial interactions. Social and Personality Psychology Compass, 5(2), 118–131. 10.1111/j.1751-9004.2010.00338.x

[bibr97-17456916211057565] Murrar S. Campbell M. R. Brauer M. (2020). Exposure to peers’ pro-diversity attitudes increases inclusion and reduces the achievement gap. Nature Human Behaviour, 4(9), 889–897. 10.1038/s41562-020-0899-5 32601460

[bibr98-17456916211057565] Newheiser A. K. Dunham Y. Merrill A. Hoosain L. Olson K. R. (2014). Preference for high status predicts implicit outgroup bias among children from low-status groups. Developmental Psychology, 50(4), 1081–1090. 24219317 10.1037/a0035054 PMC3981896

[bibr99-17456916211057565] Nisbett R. E. Wilson T. D. (1977). The halo effect: Evidence for unconscious alteration of judgments. Journal of Personality and Social Psychology, 35(4), 250–256. 10.1037/0022-3514.35.4.250

[bibr100-17456916211057565] Nolan J. M. Schultz P. W. Cialdini R. B. Goldstein N. J. Griskevicius V. (2008). Normative social influence is underdetected. Personality and Social Psychology Bulletin, 34(7), 913–923. 10.1177/0146167208316691 18550863

[bibr101-17456916211057565] Norton M. I. Sommers S. R. (2011). Whites see racism as a zero-sum game that they are now losing. Perspectives on Psychological Science, 6(3), 215–218. 10.1177/1745691611406922 26168512

[bibr102-17456916211057565] Ostrom T. M. Sedikides C. (1992). Out-group homogeneity effects in natural and minimal groups. Psychological Bulletin, 112(3), 536–552. 10.1037/0033-2909.112.3.536

[bibr103-17456916211057565] Paluck E. L. (2011). Peer pressure against prejudice: A high school field experiment examining social network change. Journal of Experimental Social Psychology, 47(2), 350–358. 10.1016/j.jesp.2010.11.017

[bibr104-17456916211057565] Paluck E. L. Porat R. Clark C. S. Green D. P. (2020). Prejudice reduction: Progress and challenges. Annual Review of Psychology, 72(1). 10.1146/annurev-psych-071620-030619 32928061

[bibr105-17456916211057565] Paluck E. L. Shepherd H. Aronow P. M. (2016). Changing climates of conflict: A social network experiment in 56 schools. Proceedings of the National Academy of Sciences, USA, 113(3), 566–571. 10.1073/pnas.1514483113 PMC4725542 26729884

[bibr106-17456916211057565] Pascoe E. A. Smart Richman L. (2009). Perceived discrimination and health: A meta-analytic review. Psychological Bulletin, 135(4), 531–554. 10.1037/a0016059 19586161 PMC2747726

[bibr107-17456916211057565] Payne B. K. (2001). Prejudice and perception: The role of automatic and controlled processes in misperceiving a weapon. Journal of Personality and Social Psychology, 81(2), 181–192. 10.1037/0022-3514.81.2.181 11519925

[bibr108-17456916211057565] Payne B. K. Vuletich H. A. Lundberg K. B. (2017). The bias of crowds: How implicit bias bridges personal and systemic prejudice. Psychological Inquiry, 28(4), 233–248. 10.1080/1047840x.2017.1335568

[bibr109-17456916211057565] Pettigrew T. F. Tropp L. R. (2006). A meta-analytic test of intergroup contact theory. Journal of Personality and Social Psychology, 90(5), 751–783. 10.1037/0022-3514.90.5.751 16737372

[bibr110-17456916211057565] Plant E. A. Baylor A. L. Doerr C. E. Rosenberg-Kima R. B. (2009). Changing middle-school students’ attitudes and performance regarding engineering with computer-based social models. Computers & Education, 53(2), 209–215. 10.1016/j.compedu.2009.01.013

[bibr111-17456916211057565] Plant E. A. Devine P. G. (1998). Internal and external motivation to respond without prejudice. Journal of Personality and Social Psychology, 75(3), 811–832. 10.1037/0022-3514.75.3.811

[bibr112-17456916211057565] Plant E. A. Devine P. G. Peruche M. B. (2010). Routes to positive interracial interactions: Approaching egalitarianism or avoiding prejudice. Personality and Social Psychology Bulletin, 36(9), 1135–1147. 20660704 10.1177/0146167210378018

[bibr113-17456916211057565] Plaut V. C. Thomas K. M. Hurd K. Romano C. A. (2018). Do color blindness and multiculturalism remedy or foster discrimination and racism? Current Directions in Psychological Science, 27(3), 200–206. 10.1177/0963721418766068

[bibr114-17456916211057565] Prentice D. Paluck E. L. (2020). Engineering social change using social norms: Lessons from the study of collective action. Current Opinion in Psychology, 35, 138–142. 10.1016/j.copsyc.2020.06.012 32746001

[bibr115-17456916211057565] Pun A. Ferera M. Diesendruck G. Kiley Hamlin J. Baron A. S. (2018). Foundations of infants’ social group evaluations. Developmental Science, 21(3), Article e12586. 10.1111/desc.12586 28703876

[bibr116-17456916211057565] Purdie-Vaughns V. Steele C. M. Davies P. G. Ditlmann R. Crosby J. R. (2008). Social identity contingencies: How diversity cues signal threat or safety for African Americans in mainstream institutions. Journal of Personality and Social Psychology, 94(4), 615–630. 10.1037/0022-3514.94.4.615 18361675

[bibr117-17456916211057565] Radke H. R. M. Kutlaca M. Siem B. Wright S. C. Becker J. C. (2020). Beyond allyship: Motivations for advantaged group members to engage in action for disadvantaged groups. Personality and Social Psychology Review, 24(4), 291–315. 10.1177/1088868320918698 32390573 PMC7645619

[bibr118-17456916211057565] Ransom J. (2020, July 6). Amy Cooper faces charges after calling police on Black bird-watcher. The New York Times. https://www.nytimes.com/2020/07/06/nyregion/amy-cooper-false-report-charge.html

[bibr119-17456916211057565] Régner I. Thinus-Blanc C. Netter A. Schmader T. Huguet P. (2019). Committees with implicit biases promote fewer women when they do not believe gender bias exists. Nature Human Behaviour, 3(11), 1171–1179. 10.1038/s41562-019-0686-3 31451735

[bibr120-17456916211057565] Rhodes M. Baron A. (2019). The development of social categorization. Annual Review of Developmental Psychology, 1, 359–386. 10.1146/annurev-devpsych-121318-084824 PMC7577394 33103119

[bibr121-17456916211057565] Richeson J. A. Nussbaum R. J. (2004). The impact of multiculturalism versus color-blindness on racial bias. Journal of Experimental Social Psychology, 40(3), 417–423. 10.1016/j.jesp.2003.09.002

[bibr122-17456916211057565] Rivera L. (2012). Hiring as cultural matching: The case of elite professional service firms. American Sociological Review, 77, 999–1022.

[bibr123-17456916211057565] Rydell R. J. McConnell A. R. Strain L. M. Claypool H. M. Hugenberg K. (2007). Implicit and explicit attitudes respond differently to increasing amounts of counterattitudinal information. European Journal of Social Psychology, 37(5), 867–878. 10.1002/ejsp.393

[bibr124-17456916211057565] Salomon K. Burgess K. D. Bosson J. K. (2015). Flash fire and slow burn: Women’s cardiovascular reactivity and recovery following hostile and benevolent sexism. Journal of Experimental Psychology: General, 144(2), 469–479. 10.1037/xge0000061 25844626

[bibr125-17456916211057565] Salter P. S. Adams G. Perez M. J. (2018). Racism in the structure of everyday worlds: A cultural-psychological perspective. Current Directions in Psychological Science, 27(3), 150–155. 10.1177/0963721417724239

[bibr126-17456916211057565] Schmader T. Bergsieker H. B. Hall W. M. (2020). Cracking the culture code. In Forgas J. Fiedler K. Crano W. (Eds.), Applications of social psychology (pp. 334–355). Routledge.

[bibr127-17456916211057565] Schmader T. Sedikides C. (2018). State authenticity as fit to environment: The implications of social identity for fit, authenticity, and self-segregation. Personality and Social Psychology Review, 22(3), 228–259. 10.1177/1088868317734080 28975851

[bibr128-17456916211057565] Schmitt M. T. Branscombe N. R. Postmes T. Garcia A. (2014). The consequences of perceived discrimination for psychological well-being: A meta-analytic review. Psychological Bulletin, 140(4), 921–948. 10.1037/a0035754 24547896

[bibr129-17456916211057565] Sears D. O. Henry P. J. (2005). Over thirty years later: A contemporary look at symbolic racism. In Zanna M. P. (Ed.), Advances in experimental social psychology (Vol. 37, pp. 95–150). Academic Press.

[bibr130-17456916211057565] Sechrist G. B. Stangor C. (2001). Perceived consensus influences intergroup behavior and stereotype accessibility. Journal of Personality and Social Psychology, 80(4), 645–654. 10.1037/0022-3514.80.4.645 11316227

[bibr131-17456916211057565] Sherman D. K. Brookfield J. Ortosky L. (2017). Intergroup conflict and barriers to common ground: A self-affirmation perspective. Social and Personality Psychology Compass, 11(12), Article e12364. 10.1111/spc3.12364

[bibr132-17456916211057565] Shields S. A. Zawadzki M. J. Johnson R. N. (2011). The impact of the Workshop Activity for Gender Equity Simulation in the Academy (WAGES–Academic) in demonstrating cumulative effects of gender bias. Journal of Diversity in Higher Education, 4(2), 120–129. 10.1037/a0022953

[bibr133-17456916211057565] Simon M. Sidner S. (2021, January 11). Decoding the extremist symbols and groups at the Capitol Hill insurrection. CNN. https://www.cnn.com/2021/01/09/us/capitol-hill-insurrection-extremist-flags-soh/index.html

[bibr134-17456916211057565] Simons R. L. Lei M.-K. Beach S. R. H. Barr A. B. Simons L. G. Gibbons F. X. Philibert R. A. (2018). Discrimination, segregation, and chronic inflammation: Testing the weathering explanation for the poor health of Black Americans. Developmental Psychology, 54(10), 1993–2006. 10.1037/dev0000511 30234347 PMC7685230

[bibr135-17456916211057565] Smith E. R. DeCoster J. (2000). Dual-process models in social and cognitive psychology: Conceptual integration and links to underlying memory systems. Personality and Social Psychology Review, 4(2), 108–131. 10.1207/s15327957pspr0402_01

[bibr136-17456916211057565] Sommers S. R. (2006). On racial diversity and group decision making: Identifying multiple effects of racial composition on jury deliberations. Journal of Personality and Social Psychology, 90(4), 597–612. 10.1037/0022-3514.90.4.597 16649857

[bibr137-17456916211057565] Stangor C. Sechrist G. B. Jost J. T. (2001). Changing racial beliefs by providing consensus information. Personality and Social Psychology Bulletin, 27, 486–496. 10.1177/0146167201274009

[bibr138-17456916211057565] Steele C. M. (1988). The psychology of self-affirmation: Sustaining the integrity of the self. In Berkowitz L. (Ed.), Advances in experimental social psychology, Vol. 21. Social psychological studies of the self: Perspectives and programs (Vol. 21, pp. 261–302). Academic Press.

[bibr139-17456916211057565] Sue D. W. Capodilupo C. M. Torino G. C. Bucceri J. M. Holder A. Nadal K. L. Esquilin M. (2007). Racial microaggressions in everyday life: Implications for clinical practice. American Psychologist, 62(4), 271–286. 17516773 10.1037/0003-066X.62.4.271

[bibr140-17456916211057565] Terrell J. Kofink A. Middleton J. Rainear C. Murphy-Hill E. Parnin C. Stallings J. (2016). Gender differences and bias in open source: Pull request acceptance of women versus men. PeerJ Computer Science, 4, Article e1733. 10.7717/peerj-cs.111

[bibr141-17456916211057565] Van Ryn M. Hardeman R. Phelan S. M. Burgess D. J. Dovidio J. F. Herrin J. Burke S. E. Nelson D. B. Perry S. Yeazel M. Przedworski J. M. (2015). Medical school experiences associated with change in implicit racial bias among 3547 students: A medical student CHANGES study report. Journal of General Internal Medicine, 30(12), 1748–1756. 10.1007/s11606-015-3447-7 26129779 PMC4636581

[bibr142-17456916211057565] Vial A. C. Brescoll V. L. Dovidio J. F. (2019). Third-party prejudice accommodation increases gender discrimination. Journal of Personality and Social Psychology, 117(1), 73–98. 10.1037/pspi0000164 30335417

[bibr143-17456916211057565] Vuletich H. A. Payne B. K. (2019). Stability and change in implicit bias. Psychological Science, 30(6), 854–862. 10.1177/0956797619844270 31050916

[bibr144-17456916211057565] Williams M. T. (2020). Microaggressions: Clarification, evidence, and impact. Perspectives on Psychological Science, 15(1), 3–26. 10.1177/1745691619827499 31418642

[bibr145-17456916211057565] Witteman H. O. Hendricks M. Straus S. Tannenbaum C. (2019). Are gender gaps due to evaluations of the applicant or the science? A natural experiment at a national funding agency. The Lancet, 393(10171), 531–540. 10.1016/s0140-6736(18)32611-4 30739688

[bibr146-17456916211057565] Yogeeswaran K. Dasgupta N. (2010). Will the “real” American please stand up? The effect of implicit national prototypes on discriminatory behavior and judgments. Personality and Social Psychology Bulletin, 36(10), 1332–1345. 10.1177/0146167210380928 20729337

[bibr147-17456916211057565] Zelevansky N. (2019, November 20). The big business of unconscious bias. The New York Times. https://www.nytimes.com/2019/11/20/style/diversity-consultants.html

